# Fabrication of pH- and Ultrasound-Responsive Polymeric Micelles: The Effect of Amphiphilic Block Copolymers with Different Hydrophilic/Hydrophobic Block Ratios for Self-Assembly and Controlled Drug Release

**DOI:** 10.1021/acs.biomac.4c01202

**Published:** 2025-03-11

**Authors:** Hong-Xiang Wei, Ming-Hsin Liu, Tzu-Ying Wang, Meng-Hsiu Shih, Jiashing Yu, Yi-Cheun Yeh

**Affiliations:** † Institute of Polymer Science and Engineering, National Taiwan University, Taipei 10617, Taiwan; ‡ Department of Chemical Engineering, National Taiwan University, Taipei 10617, Taiwan

## Abstract

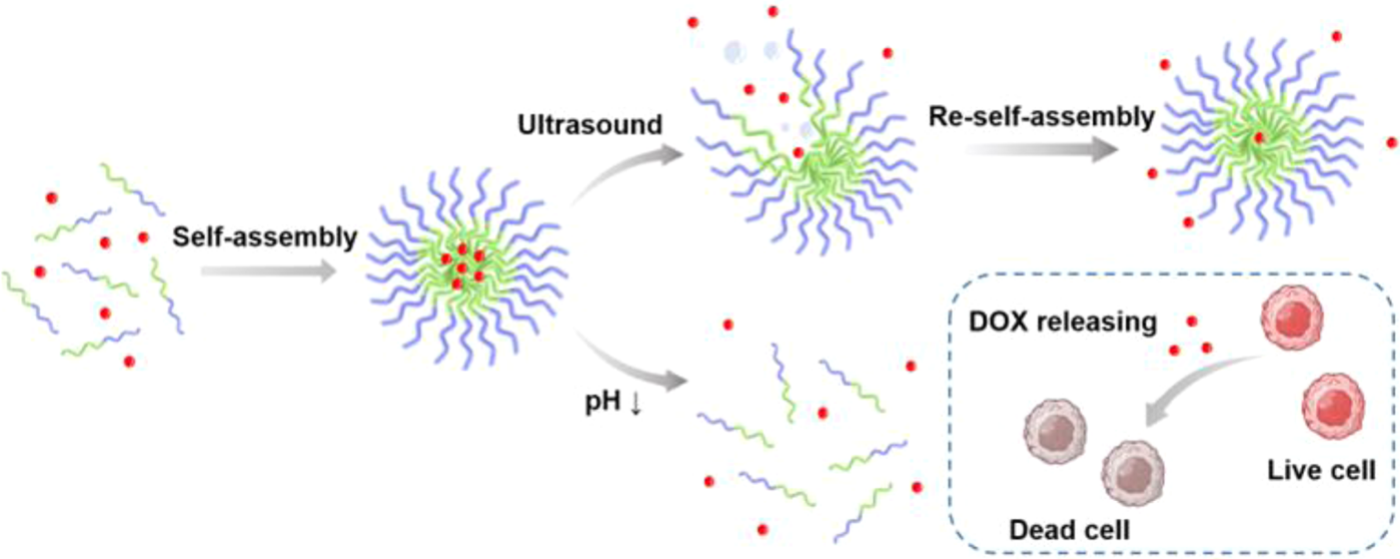

Stimuli-responsive polymeric vehicles can change their physical or chemical properties when exposed to internal or external triggers, enabling precise spatiotemporal control of drug release. Nevertheless, systematic research is lacking in preparing dual stimuli-responsive amphiphilic block copolymers with different hydrophilic/hydrophobic block ratios in forming self-assembled structures. Here, we synthesized two types of block copolymers consisting of the hydrophobic segments (i.e., pH-responsive 2-(diethylamino)ethyl methacrylate (DEA) and ultrasound-responsive 2-methoxyethyl methacrylate (MEMA)) and hydrophilic poly(ethylene glycol) methyl ether (mPEG) segments, forming mPEG_X_-*b*-P(DEA_Y_-*co*-MEMA_Z_). These amphiphilic block copolymers can self-assemble to form polymeric micelles, and their structures (e.g., size) and properties (e.g., critical vesicle concentration, stability, stimuli-responsiveness to pH and ultrasound, drug loading efficiency, and controlled drug release performance) were thoroughly investigated. *In vitro* cell studies further demonstrate that ultrasound can efficiently trigger drug release from polymeric micelles, emphasizing their potential for controlled drug delivery in therapeutic applications.

## 1 Introduction

Polymeric vehicles (e.g., polyplexes, polymeric micelles, dendrimers, and polymersomes) have become crucial components in modern drug delivery systems. Each of these vehicles exhibits unique characteristics, making them suitable for enhancing the delivery of hydrophobic drugs, nucleic acids, and other therapeutic agents.^1−4^ For example, polymeric micelles, a type of nanoscale drug delivery system, feature a core–shell structure formed through the self-assembly of amphiphilic block copolymers in aqueous solutions.^5^ The inner core of the polymeric micelles is formed by the hydrophobic segments of the copolymers, which can encapsulate poorly water-soluble drugs,^6^ antifungal agents,^7^ or polynucleotides.^8^ Meanwhile, the hydrophilic segments form the outer shell of the polymeric micelles to stabilize the micelle in biological environments.^9^ The core–shell structure of the polymeric micelles helps solubilize drugs within the core while shielding them from the aqueous environment, making polymeric micelles a promising platform for drug delivery. On the other hand, amphiphilic block copolymers can also self-assemble to form polymersomes, which possess double-layer structures to endow them with a hollow architecture.^10^ For drug delivery applications, polymersomes offer the versatility to encapsulate both hydrophobic and hydrophilic molecules, where the hydrophilic part can encapsulate water-soluble drugs^11^ or proteins,^12^ while the hydrophobic part can accommodate hydrophobic drugs,^13^ dyes,^14^ or even inorganic nanoparticles.^15^ Compared to liposomes,^16^ polymersomes exhibit better stability, greater mechanical robustness, and potential for advanced chemical functionalization and physiological applications.^17,18^

Stimuli-responsive polymeric vehicles have been demonstrated to allow the controllable payload release to enhance the therapeutic efficiency of polymeric vehicles in biomedicine.^19^ The physical or chemical properties of stimuli-responsive polymeric vehicles, especially inducing the membrane disruption in polymeric vehicles to release the encapsulated cargos, can be adjusted through stimuli. For example, polymeric micelles can be designed to be sensitive to various stimuli such as pH,^20,21^ reactive oxygen species (ROS),^22^ enzymes,^23,24^ hypoxia,^25^ redox,^26^ temperature,^27,28^ light,^29,30^ ultrasound,^31^ magnetic fields,^32^ and electric fields.^33^ Similarly, polymersomes can respond to temperature,^34^ pH,^35−37^ light,^38−41^ electric field,^42^ magnetic fields,^43,44^ ultrasound,^45^ and redox.^46^ Among these, internal stimuli (e.g., pH, redox potential, and temperature) naturally vary between healthy and diseased conditions. Among these stimuli-responsive polymeric vehicles, pH-responsive polymeric vehicles have become one of the most extensively studied systems. These polymeric vehicles can change their structure and properties (e.g., chain conformation, solubility, surface activity, and configuration) in response to pH adjustments.^47−50^ For example, Yang et al. developed pH-sensitive polymeric micelles constructed from *cis*-aconitic anhydride-modified poly(ethylene glycol)-poly(l-lysine) (PEG-pLL(CAA)) block copolymers.^51^ These polymeric micelles were stable under normal physiological pH conditions (pH 7.4), while they dissociated to release encapsulated mRNA at weakly acidic pH levels (<6.5). This system not only ensured mRNA stability at physiological pH but also enabled its release at tumor sites, promoting protein expression where it is most needed. Ultrasound-responsive polymeric vehicles are another popular class of stimuli-responsive polymeric vehicles, as ultrasound possesses numerous advantages, including noninvasiveness, precise control over timing and spatial distribution, and deep tissue penetration.^52−54^ These qualities make ultrasound an attractive trigger for enhancing drug delivery efficiency and tumor-targeting treatment. For example, Wu et al. developed polymeric micelles based on pluronic P123/F127 copolymers, which demonstrated ultrasound-triggered drug release capabilities.^55^ The release of curcumin encapsulated within these polymeric micelles was triggered by cavitation induced by ultrasound, with the extent of leakage dependent on ultrasound intensity but independent of exposure duration. In another example, Hammer et al. reported the release of molecules from polymersomes made from poly(ethylene oxide)-*b*-polybutadiene (PEO-*b*-PBD) copolymers when exposed to ultrasound.^14^ They found that ultrasound at specific intensities causes significant leakage of the fluorescent dye (i.e., 8-aminonaphthalene-1, 3, 6-trisulfonic acid (ANTS)) from the vesicle core due to acoustic cavitation. The extent of leakage depends on the time and intensity of the ultrasound exposure.

Given that the tumor microenvironment is complex, polymeric vehicles that respond to a single stimulus often struggle to inhibit cancer growth effectively. Consequently, the design and utilization of multistimuli-responsive polymeric vehicles have gained increasing attention. Among the various stimuli, pH and ultrasound have emerged as prevalent choices due to their ability to be easily adjusted and then enhance the efficacy of cancer treatment. For example, Yildirim et al. reported a dual-stimuli-responsive polymeric nanoparticle system synthesized from 3,4-dihydro-2H-pyran (DHP)-protected HEMA [2-((tetrahydro-2H-pyran-2-yl)oxy)ethyl methacrylate (THP-HEMA)] and 2-(dimethylamino)ethyl methacrylate (DMAEMA).^56^ The THP functional segments on THP-HEMA reacted to ultrasound treatment because of their low glass transition temperature and lack of crystallinity, which increased molecular mobility and sensitivity to ultrasound. Under acidic conditions, complete protonation of the DMAEMA groups resulted in nanoparticle disassembly at endosomal pH levels. The polymeric nanoparticles exhibited an enhanced drug release rate under acidic conditions and upon ultrasound treatment, highlighting the controllability and enhanced therapeutic potential of multistimuli-responsive polymeric nanoparticles. However, to the best of our knowledge, the current research has not explored the systematic investigation of dual stimuli-responsive amphiphilic block copolymers prepared using the same feed ratios of precursors but with varying PEG chain lengths, which resulted in different hydrophilic/hydrophobic block ratios for the formation of polymeric vehicles.

**Scheme 1:**
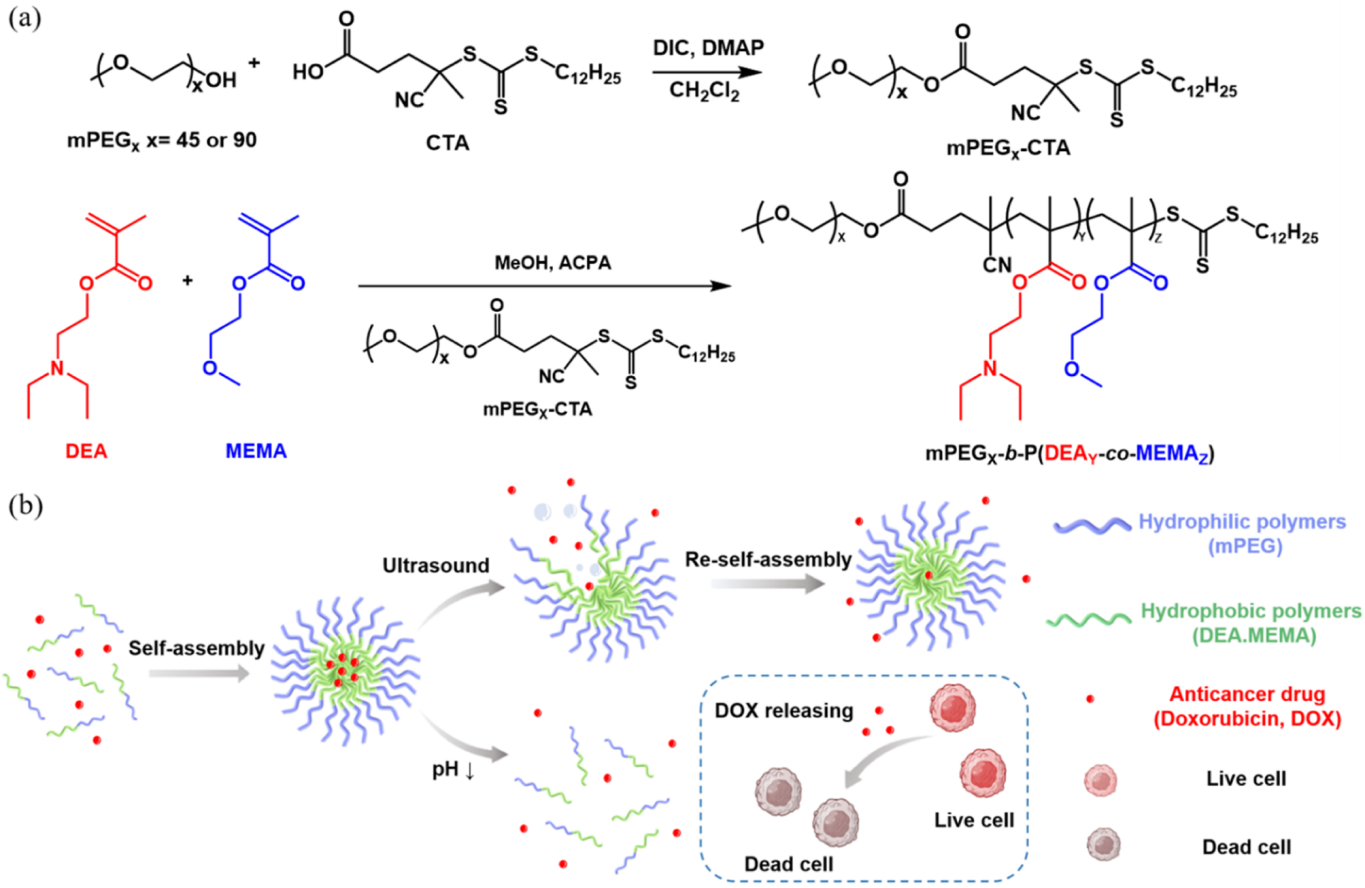
(a) Synthetic Schemes of mPEG_X_-*b*-P(DEA_Y_-*co*-MEMA_Z_) Block Copolymers. (b) Schematic Illustrations of the Polymeric Vehicle Formation and the pH- and Ultrasound-Triggered Drug Release to Kill Cancer Cells. mPEG= Poly(ethylene glycol)methyl ether; CTA= 4-Cyano-4-[(dodecylsulfanylthiocarbonyl)sulfanyl]pentanoic Acid; DIC= Diisopropyl carbodiimide; DMAP= 4-Dimethylaminopyridine; DEA= 2-(Diethylamino)ethyl methacrylate; MEMA= 2-Methoxyethyl methacrylate; ACPA= 4,4′-Azobis(4-cyanovaleric Acid).

Here, we synthesized two types of dual-responsive (i.e., pH and ultrasound) block copolymers using the same feed ratios of precursors but with varying PEG chain lengths to fabricate polymeric vehicles through self-assembly. The block copolymers were synthesized through reversible addition–fragmentation chain-transfer (RAFT) polymerization to consist of the hydrophobic segments (i.e., pH-responsive 2-(diethylamino)ethyl methacrylate (DEA) and ultrasound-responsive 2-methoxyethyl methacrylate (MEMA)) and hydrophilic poly(ethylene glycol) methyl ether (mPEG) segments, forming mPEG_X_-*b*-P(DEA_Y_-*co*-MEMA_Z_) (Scheme 1a). DEA is a widely used pH-sensitive functional group as the protonation of the tertiary amine groups within DEA disrupts the self-assembled structures.^57,58^ On the other hand, ultrasound waves generally create high-frequency mechanical vibrations that influence the mobility of the hydrophobic segments in block copolymers due to cavitation, thereby enhancing chain movement. When exposed to ultrasound irradiation, these polymeric structures undergo rapid disruption and reassembly, resulting in smaller structural sizes. Some segments (e.g., 2-(tetrahydrofuranyloxy)ethyl methacrylate (TMA)^57,59^ and MEMA^60,61^) are commonly utilized to construct ultrasound-responsive polymeric structures, and MEMA was used in this study. The feed ratios of precursors (i.e., DEA, MEMA, chain transfer agent coupled with mPEG (mPEG-CTA), and initiator) were kept consistent in synthesizing the block copolymers, while mPEG with different chain lengths (i.e., mPEG_45_ and mPEG_90_) were used. These two block copolymers, along with two control groups (i.e., ultrasound-responsive mPEG_X_-*b*-PMEMA_Z_ and pH-responsive mPEG_X_-*b*-PDEA_Z_ block copolymers), were used for self-assembly. The structures and properties of these polymeric vehicles were systematically compared, including the size, critical vesicle concentration (CVC), drug encapsulation efficiency, stability, and stimuli-responsiveness toward pH and ultrasound. These stimuli-responsive mPEG_X_-*b*-P(DEA_Y_-*co*-MEMA_Z_) polymeric vehicles were further applied as drug carriers to perform controlled drug release for cancer therapy (Scheme 1b).

## 2 Materials and Methods

### 2.1 Materials

Poly(ethylene glycol) methyl ether 2000 (mPEG_45_), poly(ethylene glycol) methyl ether 4000 (mPEG_90_), and 2-(diethylamino)ethyl methacrylate (DEA) were purchased from TCI. Diisopropyl carbodiimide (DIC), 4,4′-azobis(4-cyanovaleric acid) (ACPA), phosphotungstic acid, and doxorubicin hydrochloride (DOX·HCl) were purchased from Morchem. 4-Dimethylaminopyridine (DMAP) was purchased from Fluorochem. 1-Dodecanethiol and p-toluenesulfonyl chloride were purchased from Acros Organics. Carbon disulfide, acetonitrile, and fetal bovine serum (FBS) were purchased from Fisher Scientific. 2-Methoxyethyl methacrylate (MEMA) and pyrene were purchased from BLD Pharmate. Potassium hydroxide was purchased from Xilong Scientific co. ltd. Dichloromethane was purchased from Reagents Duksan. Ethyl ether (anhydrous), hexane, methanol, and ethanol were purchased from ECHO Chemical co. ltd. Acetone was purchased from Everdine technology co. ltd. Tetrahydrofuran was purchased from Macron. Ethyl acetate was purchased from Uni-Onward corp. Phosphate buffered saline (PBS) was purchased from Biomate. Dulbecco’s modified Eagle medium high glucose (DMEM-HG) and antibiotic antimycotic solution (penicillin/streptomycin/amphotericin, PSA) were purchased from Capricorn Scientific. Trypsin-EDTA (10x) was purchased from Biological Industries. Cell Counting Kit-8 (CCK-8) was purchased from Elabscience. LIVE/DEAD Viability/Cytotoxicity Kit was purchased from Invitrogen.

### 2.2 Instruments

The ^1^H nuclear magnetic resonance (NMR) spectra of compounds were acquired using a Bruker Avance III HD-600 MHz NMR spectrometer, with samples dissolved in CDCl_3_. The electrospray ionization mass spectrometry (ESI-MS) analysis of compounds was performed with a QSTAR XL mass spectrometer. Fourier-transform infrared (FTIR) spectra were obtained using a PerkinElmer Spectrum Two instrument. Gel permeation chromatography (GPC) was conducted with the machine (Waters) equipped with the 2410 refractive index detector and Styragel columns of HR2 and HR4E for the organic phase (THF). For the aqueous phase, the GPC machine (Enshine SUPER CO-150) was used, which was equipped with the Waters 2414 refractive index detector (RI) and TSKgel G5000PW column. The samples were dissolved in water, and P-82 (Pullulan) was used as the standard. The flow rate of GPC measurement was set at 0.6 mL/min. The ultrasonic responsive experiments were conducted using an ultrasound machine (US-751, ITO Co., Ltd.). Temperature changes were recorded using a touch-sensitive paperless recorder (VM7000 WH10, Well). The size distribution and zeta potential of the polymeric micelles were determined through dynamic light scattering (DLS) analysis using the ELSZ-2000 analyzer. The morphology of polymeric micelles was characterized using transmission electron microscopy (TEM, Hitachi H7100, 75 kV) and cryo-TEM (FEI Tecnai G2 F20 TWIN). Absorbance was measured using a UV–vis spectrophotometer (Jasco V-650).

### 2.3 Synthesis of 4-Cyano-4-[(dodecylsulfanylthiocarbonyl)sulfanyl] pentanoic Acid

4-Cyano-4-[(dodecylsulfanylthiocarbonyl)sulfanyl] pentanoic acid (CDTPA) was synthesized according to the reported process.^62^ In a single-neck round-bottom flask, 1-dodecanethiol (19.12 g, 0.094 mol) was dissolved in 80 mL of a 10% ethanol aqueous solution. Potassium hydroxide (KOH) (3.09 g, 0.055 mol) was then added to the solution with vigorous stirring. After KOH was fully dissolved, carbon disulfide (7.17 g, 0.095 mol) was added dropwise to the mixture and stirred for 3 h. Subsequently, p-toluenesulfonyl chloride (8.78 g, 0.048 mol) dissolved in 5 mL of dichloromethane (DCM) was slowly added into the mixture under cooling conditions. After stirring at room temperature for 5 h, the mixture was extracted four times with 30 mL of DCM. The organic phases were combined and washed three times with saturated sodium bicarbonate solution and two times with deionized water. The obtained organic solution was dried with anhydrous sodium sulfate, the solids were then filtered out, and the excess solvent was removed under a rotary evaporator to obtain the intermediate (bis(dodecylmercaptothiocarbonyl) disulfide) (Compound 1) (22 g, yield= 83%) (Scheme S1).

Compound 1 (12 g, 0.0215 mol) was dissolved in 200 mL of ethyl acetate. 4,4′-azobis(4-cyanovaleric acid) (6.95 g, 0.0235 mol) was added to the solution. The mixture was stirred at 80 °C for 12 h. Subsequently, the product CDTPA was concentrated to remove the excess solvent of ethyl acetate and precipitated by *n*-hexane. CDTPA as a chain transfer agent (CTA) in the RAFT reaction was obtained as a yellow solid (16.8 g, yield= 93%) (Scheme S1).

### 2.4 Synthesis of mPEG_X_-CTA

CTA was reacted with poly(ethylene glycol) methyl ether (mPEG) to form mPEG-CTA based on the reported procedure.^63^ Briefly, mPEG_X_-CTA was synthesized via a Steglich esterification reaction, where CTA reacted with mPEG_*x*_ (*X* = 45 or 90). During the reaction process, diisopropyl carbodiimide (DIC) served as the coupling reagent, and 4-dimethylaminopyridine (DMAP) acted as the catalyst. mPEG_45_ (2 g, 1 mmol) or mPEG_90_ (4 g, 1 mmol), along with CTA (807.32 mg, 2 mmol), DIC (252.4 mg, 2 mmol), and DMAP (24.43 mg, 0.2 mmol), were added to a single-neck round-bottom flask containing DCM (30 mL). The flask was sealed with a rubber septum, and the esterification reaction proceeded with stirring at room temperature for 24 h. Subsequently, the mPEGx-CTA was collected by precipitating the reaction mixture in cold diethyl ether three times. The resulting yields were 79% for mPEG_45_-CTA (1.9 g) and 81% for mPEG_90_-CTA (3.6 g).

### 2.5 Syntheses of mPEG_X_-*b*-P(DEA_Y_-*co*-MEMA_Z_) Block Copolymers

An alumina column was utilized to remove the monomethyl ether hydroquinone (MEHQ) inhibitor from 2-(diethylamino)ethyl methacrylate (DEA) and 2-methoxyethyl methacrylate (MEMA). DEA (276 mg, 1.76 mmol), MEMA (254 mg, 1.76 mmol), mPEG_45_-CTA (180 mg, 0.09 mmol) or mPEG_90_-CTA (360 mg, 0.09 mmol), 4,4′-azobis(4-cyanovaleric acid) (ACPA) (3 mg, 0.011 mmol), and MeOH (2 mL) were combined in the polymerization tube (Scheme 1a). The reaction tube was degassed by three freeze–pump–thaw cycles before placing it in an oil bath at 70 °C for 24 h. The obtained crude product was concentrated, precipitated with ethyl ether and water, and subsequently filtered. The resulting solid was dried in a vacuum oven to yield the product termed mPEG_X_-*b*-P(DEA_Y_-*co*-MEMA_Z_).

The syntheses of mPEG_45_-*b*-PMEMA_Z_ and mPEG_45_-*b*-PDEA_Y_ block copolymers followed a similar procedure. Before use, MEMA and DEA were treated with an alumina column to eliminate the MEHQ inhibitor. In each case, MEMA (507 mg, 3.52 mmol) or DEA (553 mg, 3.52 mmol), along with mPEG_45_-CTA (180 mg, 0.09 mmol), 4,4′-azobis(4-cyanovaleric acid) (ACPA) (3 mg, 0.011 mmol), and MeOH (2 mL) were added to a polymerization tube. The tube was evacuated three times using liquid nitrogen and a pump. Subsequently, it was placed in an oil bath at 70 °C for 24 h to allow for reaction. The resulting crude product was concentrated, precipitated with ethyl ether (for mPEG_45_-*b*-PMEMA_Z_) or hexane (for mPEG_45_-*b*-PDEA_Y_) and water, and then filtered. The solid was dried in a vacuum oven to obtain the products, mPEG_45_-*b*-PMEMA_Z_ and mPEG_45_-*b*-PDEA_Y_ (Scheme S2).

### 2.6 Self-Assembly of Block Copolymers to Form Polymeric Vehicles

The self-assembly of block copolymers was carried out using the solvent-switching method. The block copolymer was dissolved in THF to form a solution with a concentration of 2 mg/mL. Subsequently, distilled water with a double volume was slowly added to the solution by a syringe pump with a flow rate of 0.1 mL/min at room temperature (R.T.). Lastly, the mixture solution was dialyzed for 1 day using a dialysis bag (MWCO= 6000–8000) to remove THF.

### 2.7 Critical Vesicle Concentration (CVC) of Polymeric Vehicles

To determine the critical vesicle concentration (CVC) of the polymeric vehicles, pyrene (3.0 mg, 15 μmol) was dissolved in acetone (25 mL) to prepare a pyrene solution (6 × 10^–5^ M). Subsequently, pyrene solution (1 μL) was dispensed into each centrifuge tube and evaporated overnight at room temperature (R.T.). The polymeric vehicle solution was then serially diluted using the semidilution method and added to the centrifuge tubes containing the pyrene solution. Fluorescence intensity measurements were conducted using a microplate reader (Synergy H1, BioTek) with excitation at 334 nm and emission ranging from 370 to 450 nm. The intensity of each sample was evaluated at 384 nm. By plotting the intensity values against the logarithm of the concentration of each polymeric vehicle sample, the intersection point of the two linear regression lines calculated in the linear portion of the figure provided the CVC.^64,65^

### 2.8 Morphology of Polymeric Vehicles

The polymeric vehicle solution was dropped onto a copper grid for TEM investigation. After the solution was absorbed into the copper grid, a phosphotungstic acid solution (2%) was added. After 15 s, the phosphotungstic acid solution was absorbed, and the sample was then placed into the TEM instrument (Hitachi H7100, 75 kV).^66^ For cryo-TEM sample preparation, a Vitrobot Mark IV (Thermo Fisher Scientific) was utilized under precisely controlled conditions of 4 °C and 100% humidity. Approximately 4 μL of the purified sample was applied to glow-discharged Holey Carbon Film and incubated for 10 s. The grids were subsequently vitrified through rapid plunging into liquid ethane, which was cooled by liquid nitrogen. The grids were then stored in liquid nitrogen until further imaging.

### 2.9 Drug Encapsulation of Polymeric Micelles

The block copolymer (20 mg) and doxorubicin hydrochloride (DOX·HCl) (5 mg) were dissolved in THF (10 mL) to prepare a copolymer/DOX mixed solution. In a dark environment, deionized water (20 mL) was added dropwise into the solution at a 0.1 mL/min flow rate via a syringe pump and stirred for 12 h to form DOX-loaded polymeric micelles. The mixture solution was then dialyzed for 4 h using a dialysis bag (MWCO= 6000–8000), and the deionized water was replaced every hour to remove THF and unloaded DOX to obtain DOX-loaded polymeric micelles. DOX·HCl was dissolved in PBS solution to prepare DOX solutions with different concentrations ranging from 0.78 to 100 μg/mL.

The absorbance at 480 nm of the DOX solutions was recorded using the UV–vis spectrometer for plotting the calibration curve. Similarly, the absorption of DOX-loaded polymeric micelles after dialysis was measured using the UV–vis spectrometer. The drug loading efficiency (DLE) and drug loading content (DLC) were calculated by the calibration curve and the following equations^67^
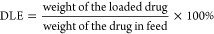
1
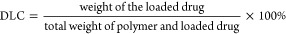
2

### 2.10 *In Vitro* Drug Release Experiments of Polymeric Micelles

The *in vitro* drug release experiment involved encapsulating DOX-loaded polymeric micelles (3 mL) in a dialysis bag (MWCO= 6000–8000 Da) placed in a beaker filled with PBS. For ultrasound-triggered drug release, the polymeric micelle samples were irradiated with ultrasound at different power intensities (i.e., 1 MHz with power densities of 0.5, 0.75, or 1 W/cm^2^) for 3 min before dialysis. For pH-triggered drug release, the dialysis was conducted against PBS buffer (30 mL) with different pH values (i.e., pH 7.4, 6.5, or 5.5) at 37 °C with stirring at 100 rpm. The absorption spectrum of the external liquid from the dialysis bag was measured at specific time intervals. The release curve was then constructed using the DOX calibration curve.

### 2.11 Cell Cultures

Mouse breast cancer cells (4T1) and mouse fibroblast cells (L929) were cultured in DMEM high glucose (DMEM-HG) medium supplemented with 10% fetal bovine serum (FBS) and 1% penicillin/streptomycin/amphotericin. Cells were incubated at 37 °C with 5% CO_2_ in a humid incubator. The medium was replenished every 2–3 days until cell confluence reached over 90% in 10 cm culture dishes. Upon reaching this confluence, the medium was aspirated, and the cells were washed with 4–5 mL of PBS. Subsequently, trypsin-EDTA diluted in PBS (0.05%, 2 mL) was added to the dishes, and incubation proceeded for 5 min at 37 °C. The enzyme reaction was halted by adding DMEM-HG medium with FBS and centrifugation for 5 min. Following the removal of the supernatant, cells were resuspended in the medium. The number of suspended cells was calculated using a hemocytometer (Suremark Hand Tally Counter SQ-3338).

### 2.12 *In Vitro* Cytotoxicity of Polymeric Micelles

The cytotoxicity of the Dox-free and Dox-loaded polymeric micelles on cells was assessed using the CCK8 assay. A volume of 100 μL containing 10,000 cells per well was dispensed into 96-well plates. Following a 24-h incubation period at 37 °C, the culture media were replaced with medium-containing polymeric micelles at various concentrations for an additional 24 h. Then, the medium was aspirated, and CCK8 solution in the medium (with a ratio of CCK8 to the medium of 1:10) (110 μL) was added, followed by a 1-h incubation period. Afterward, the CCK8 solution (110 μL) that reacted with the cells was transferred to another 96-well plate, while the initial plate containing cells was replenished with fresh medium for an additional day of culture. The cytotoxicity of polymeric micelles was determined by measuring the absorbance increase at 450 nm via a microplate reader (SpectraMax i3x). This process was repeated after 1 day to assess toxicity on day 2.

### 2.13 *In Vitro* Controlled Drug Release Performance of Polymeric Micelles

4T1 cells were seeded in a 96-well plate and precultured for 1 day. Subsequently, 100 μL of medium with 0.5 μg/mL DOX-loaded polymeric micelles was added and further incubated at 37 °C for 2 h. The ultrasound-treated groups (DM2-US and DM4-US) were subjected to a 1 MHz ultrasound at 1 W/cm^2^ intensity for 3 min. Following a one-day incubation period, cell viability was assessed using the CCK8 assay to evaluate the effects of ultrasound exposure. The following day, the CCK8 assay was repeated to evaluate cell viability at day 2 after treatment. To confirm the cell viability post-ultrasound treatment, Live/Dead cell viability staining was performed. After washing the wells once, a solution containing Calcein-AM (1:500) and ethidium homodimer-1 (1:2000) in PBS solution (1 mL) was added to the wells. Following a 15-min incubation period, the wells were rewashed. The Live/Dead staining was observed and recorded using a fluorescence microscope (IX71, OLYMPUS, Japan).

### 2.14 Statistical Analysis

Experimental data are presented as the mean ± standard deviation (SD). All statistical analyses were conducted using Prism version 8.01 software. The statistical significance was evaluated through one-way analysis of variance (ANOVA), followed by post-hoc tests to compare group differences. Results with *p* values greater than 0.05 were considered nonsignificant (n.s.). Significant results were indicated as follows: *p < 0.05, **p < 0.01, and ***p < 0.001.

## 3 Results and Discussion

### 3.1 Characterizations of 4-Cyano-4-[(dodecylsulfanylthiocarbonyl)sulfanyl] pentanoic Acid

4-Cyano-4-[(dodecylsulfanylthiocarbonyl)sulfanyl] pentanoic acid, as a chain transfer agent (CTA) in the RAFT reaction, was synthesized through a two-step process (Scheme S1).^62^ The structure of compound 1 in Scheme S1 was confirmed via ^1^H NMR analysis, which exhibited characteristic peaks at 0.89, 1.28, 1.70, and 3.31 ppm, corresponding to the hydrogen chemical shifts of the terminal methyl group, adjacent terminal methyl group, adjacent C–S bond, and carbon–sulfur bond, respectively (Figure S1a). In addition, the peak with *m*/*z* = 555.07 in the ESI-MS spectrum of compound 1 confirmed the successful synthesis of compound 1 (Figure S1b). Subsequently, compound 1 reacted with 4,4′-azobis(4-cyanovaleric acid) (ACPA) to obtain 4-Cyano-4-[(dodecylsulfanylthiocarbonyl)sulfanyl] pentanoic acid (CTA). The ^1^H NMR spectrum of CTA displayed characteristic peaks at 0.89, 1.27, 1.73, 1.89, 2.44, 2.69, and 3.36 ppm, corresponding to specific hydrogen atoms in the CTA structure (Figure S2a). Additionally, the structure of CTA was also confirmed via ^13^C NMR analysis. The carbon signal at approximately 23 ppm corresponds to the terminal methyl carbon. The carbons along the 12-carbon chain were observed at 27–30 and 37 ppm. The carbon of the C=S bond, −COOH group, and −CN group were observed at 218, 176, and 119 ppm, respectively (Figure S2b). Subsequently, the signal corresponding to CTA (*m*/*z* = 404.2) was evident in the ESI-MS spectrum (Figure S2c). Furthermore, characteristic bands at 2260 and 1706 cm^–1^ in the FTIR spectrum of CTA indicated stretching vibrations of C≡N and C=O, respectively, and bands at 1050 and 576 cm^–1^ were attributed to stretching vibrations of C=S and C–S, respectively. The above spectral analyses verified that CTA was successfully synthesized.

### 3.2 Characterizations of mPEG_X_-CTA

CTA was further reacted with poly(ethylene glycol) methyl ether (mPEG) with different molecular weights to form mPEG_X_-CTA (*X* = 45 or 90) via a Steglich esterification reaction.^63^ Analyzing the ^1^H NMR spectra of mPEG_45_-CTA and mPEG_90_-CTA revealed the integral value of the methylene protons of the mPEG_X_ backbone (around 3.7 ppm), representing the number of hydrogens within the repeated ethylene glycol unit in mPEG_X_ (Figure S3). It was found that the ratio of integral values methylene protons of the PEG backbone for mPEG_90_-CTA and mPEG_45_-CTA was about 2, confirming the correctness of the structures of mPEG_45_-CTA and mPEG_90_-CTA.

We estimated the molecular weights for the macroCTA samples using NMR, and also ran the GPC analysis for both mPEG and macroCTA samples using different solvents as eluents (i.e., THF and water) (Table S1). From the GPC traces of the polymers (Figure S4), the number-average molecular weight (*M*_n_), weight-average molecular weight (*M*_w_), and molar-mass dispersity (*Đ*M) could be determined. The results indicated that the *M*_n_ values obtained from GPC in the aqueous phase were closer to the estimates derived from NMR. In contrast, the *M*_n_ values obtained from GPC in the organic phase (THF) were higher than those estimated by NMR, including for the mPEG samples. This difference may be attributed to the larger molar volume of PEG in THF compared to water,^68^ where the expanded conformation of PEG in THF could be due to the weaker intermolecular forces. Since GPC measures the time a polymer takes to elute through a column based on its hydrodynamic volume, the more expanded PEG chains in THF present a larger hydrodynamic volume, resulting in later elution times and a higher molecular weight. Overall, the *M*_n_ values for mPEG_45_-CTA and mPEG_90_-CTA were 2886 (*Đ*M= 1.03) and 4522 (*Đ*M= 1.09) g/mol, respectively, determined by GPC in the aqueous phase.

The functional groups on the materials (i.e., mPEG_45_, mPEG_90_, CTA, mPEG_45_-CTA, and mPEG_90_-CTA) were further characterized using an FTIR spectrometer (Figure S5). In the FTIR spectrum of the materials, the obvious peak at 1085 cm^–1^ corresponds to C–O stretching vibration, while stretching and bending vibrations of methylene were observed at 2887 and 1467 cm^–1^, respectively. The band of C=O stretching vibration appearing at 1752 cm^–1^ was attributed to the formation of an ester bond, which was found on the mPEG_45_-CTA and mPEG_90_-CTA. Furthermore, compared to CTA, a decrease in signal intensity of the C≡N functional group was observed in mPEG_45_-CTA and mPEG_90_-CTA, possibly due to the more considerable molecular weight of the main chain.^69^ These peaks were evident in the successful syntheses of mPEG_45_-CTA and mPEG_90_-CTA.

### 3.3 Characterizations of Block Copolymers

**Figure 1:**
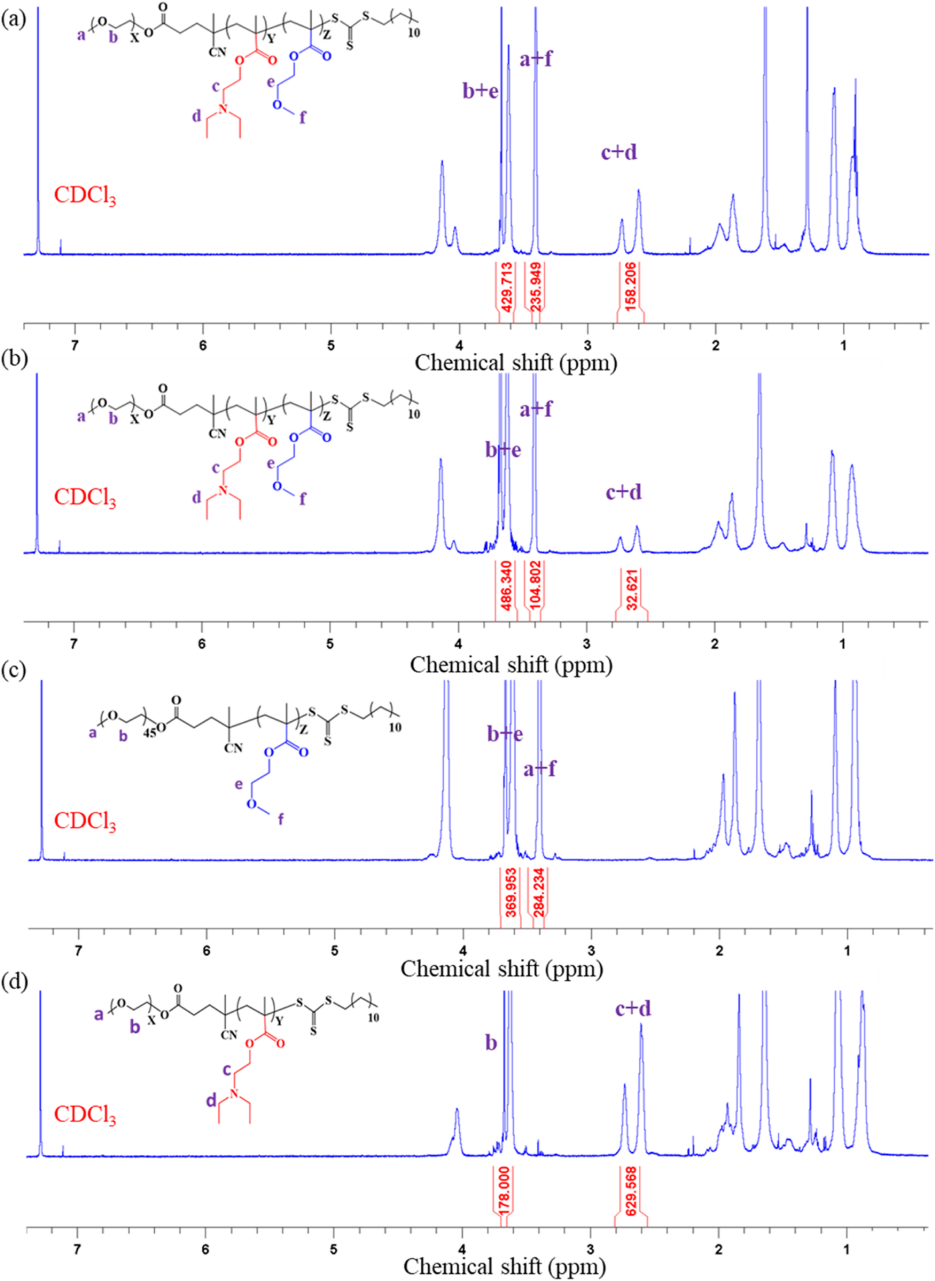
^1^H NMR spectra of (a) mPEG_45_-*b*-P(DEA_26_-*co*-MEMA_49_) (DM2), (b) mPEG_90_-*b*-P(DEA_5_-*co*-MEMA_26_) (DM4), (c) mPEG_45_-*b*-PMEMA_91_ (M2), and (d) mPEG_45_-*b*-PDEA_105_ (D2) block copolymers.

The mPEG_X_-*b-*P(DEA_Y_-*co*-MEMA_Z_) block copolymers with different PEG chain lengths were synthesized to form polymeric vehicles, where the feed ratios of precursors (i.e., DEA, MEMA, mPEG_45_-CTA or mPEG_90_-CTA, and initiator) were kept consistent. The 2-(diethylamino)ethyl methacrylate (DEA) and 2-methoxyethyl methacrylate (MEMA) in the polymeric structures were utilized as pH-responsive and ultrasonic-responsive segments, respectively. The block copolymer, mPEG_45_-*b*-P(DEA_26_-*co*-MEMA_49_) (DM2), with 26 repeat units of DEA and 49 repeat units of MEMA, as determined by ^1^H NMR spectrum through the calculation of the integral areas between peaks at 2.58–2.79 ppm of DEA and proton signals at 3.38–3.41 ppm and 3.59–3.71 ppm originating from mPEG and MEMA, respectively (Figure 1a). According to the GPC characterizations, *M*_n_ and *M*_w_ of mPEG_45_-*b*-P(DEA_26_-*co*-MEMA_49_) were 14,124 and 15,617 g/mol, respectively, with *Đ*M of 1.10 (Figure S6a). The repeating units of DEA and MEMA on mPEG_90_-*b*-P(DEA_5_-*co*-MEMA_26_) (DM4) were found to be 5 and 26, respectively (Figure 1b). The *M*_n_ and *M*_w_ of mPEG_90_-*b*-P(DEA_5_-*co*-MEMA_26_) were calculated as 11,253 and 14,400, respectively, with *Đ*M of 1.27 from the GPC spectrum (Figure S6b). The significant difference in the hydrophilic/hydrophobic block ratio between D2 (0.17) and D4 (0.85) can be attributed to the high molecular weight of mPEG_90_ in D4 (Table 1). Additionally, mPEG_90_ contributed to a steric effect, which reduced the amounts of DEA and MEMA in DM4 compared to DM2 during RAFT polymerization. The mPEG_45_-*b*-PMEMA_91_ (M2) and mPEG_45_-*b*-PDEA_105_ (D2) copolymers were synthesized and characterized as controlled groups in this study (Figures 1c,d, and S6c,d). The detailed synthetic conditions in feed ratios of precursors, the molecular weights, and the hydrophilic/hydrophobic block ratios for the four block copolymers were summarized in Table 1. It should be noted that the difference in *M*_n_ values based on NMR and GPC for polymers could be due to several factors such as sample preparation and conditions, GPC calibration standards, and NMR peak analysis, with GPC providing polydispersity insights that NMR may not directly reflect, influencing comparative results.

**Table 1:** Synthetic Conditions in Feed Ratios of Precursors and the GPC Analysis for the Block Copolymers, and Characterizations of Polymeric Vehicles

block copolymer	feed ratio [DEA]:[MEMA]: [CTA]:[initiator]	composition^a^	hydrophilic/hydrophobic block ratio^b^	*M*_n_^c^	*M*_n_^d^	*M*_w_^d^	*Đ*M^d^	CVC (μg/mL)
DM2	20:20:1:0.125	mPEG_45_-*b*-P(DEA_26-_*co*-MEMA_49_)	0.17	14,265	9861	13,192	1.34	3.8
DM4	20:20:1:0.125	mPEG_90_-*b-*P(DEA_5_-*co*-MEMA_26_)	0.85	8634	8593	10,731	1.25	4.4
M2	0:40:1:0.125	mPEG_45_-*b*-PMEMA_91_	0.15	15,099	14,345	17,478	1.21	2.8
D2	40:0:1:0.125	mPEG_45_-*b*-PDEA_105_	0.10	21,432	14,590	17,324	1.19	3.4

a The block copolymer composition was determined by ^1^H NMR spectra.

b The hydrophilic/hydrophobic block ratio was calculated using the block copolymer composition determined by ^1^H NMR spectra.

c *M*_n_ was calculated using ^1^H NMR spectra.

d *M*_n_, *M*_w_, and *Đ*M were determined by GPC.

### 3.4 Self-Assembly of Block Copolymers and Stimuli-Responsiveness of Polymeric Vehicles

The self-assembly of the block copolymers was conducted by using the solvent-switch method. The critical vesicle concentration (CVC) of each sample was calculated by measuring their fluorescence intensity (Figure S7).^64,65^ The intensity values corresponding to different concentrations of block copolymers were plotted in Figure S8, showing that the CVC of DM2, DM4, M2, and D2 block copolymers were 3.8, 4.4, 2.8, and 3.4 μg/mL, respectively (Table 1). In general, the CVC of the block copolymers is influenced by their hydrophobicity, and polymeric vehicles are formed when the hydrophobicity reaches a sufficient level.^70^ Thereafter, longer hydrophobic blocks generate stronger intermolecular interactions in aqueous environments, facilitating the formation of polymeric vehicles at lower concentrations. Here, the DM2 exhibited a lower CVC than the DM4, possibly due to the DM2 possessing a lower hydrophilic-to-hydrophobic block ratio in the structure. On the other hand, with the same PEG chain length in the block copolymer, the lower CVC of the M2 compared to the D2 may be attributed to the MEMA segments more effectively stabilizing the hydrophobic core of the polymeric vehicles than the DEA segments with diethyl chains which can produce the steric hindrance.

**Figure 2:**
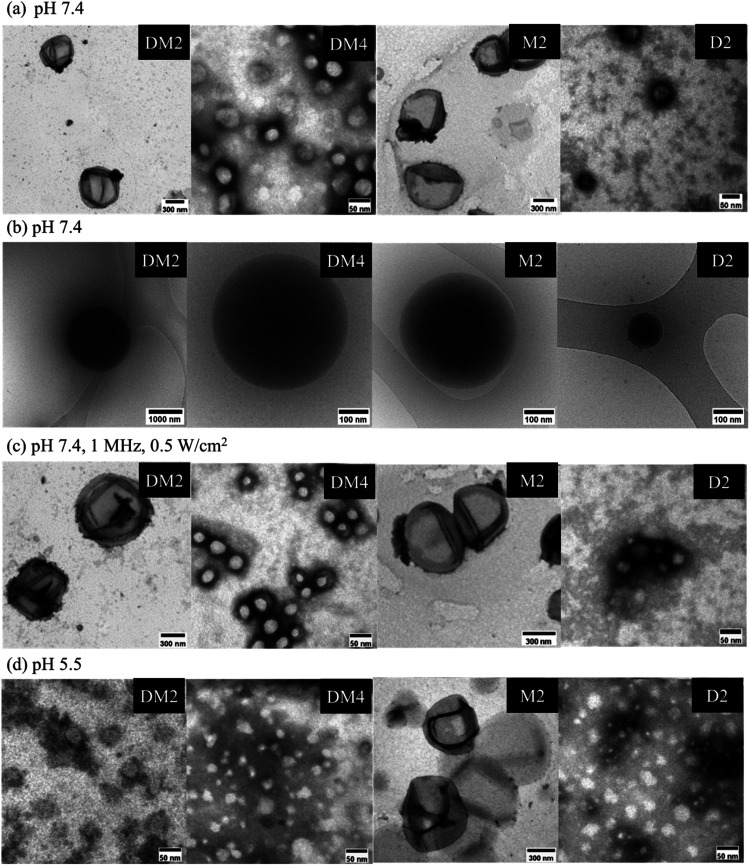
Morphology of polymeric micelles under different conditions. (a) TEM and (b) cryo-TEM images of polymeric micelles at pH 7.4. (c) TEM images of polymeric micelles in the presence of ultrasound (1 MHz, 0.5 W/cm^2^) at pH 7.4. (d) TEM images of polymeric micelles at pH 5.5.

The morphology of the polymeric vehicles was revealed using TEM, showing that the polymeric vehicles possessed a spherical structure (Figure 2a). The diameters of DM2, DM4, M2, and D2 polymeric vehicles determined by TEM images were 670 ± 70, 70 ± 10, 620 ± 100, and 70 ± 10 nm, respectively (Table 2). Cryo-TEM was further employed to investigate these polymeric vehicles, showing these amphiphilic block copolymers self-assemble to form polymeric micelles (Figure 2b).

**Table 2:** Characterizations of Polymeric Micelles

polymeric micelles	diameter of polymeric micelles (nm)^a^	hydrodynamic diameter of polymeric micelles (nm)^b^	zeta potential of polymeric micelles (mV)
DM2	670 ± 70	720 ± 260	+26.3 ± 0.7
DM4	70 ± 10	350 ± 140	+28.0 ± 0.5
M2	620 ± 100	740 ± 290	–42.4 ± 0.2
D2	70 ± 10	120 ± 60	+24.5 ± 0.9

a Diameters of polymeric micelles calculated by analyzing the TEM images.

b Hydrodynamic diameters of polymeric micelles determined through DLS.

The observed size differences between DM2 and DM4 polymeric micelles can be attributed to the varying PEG lengths in the copolymer structures. Longer PEG chains increase the hydrophilic weight fraction, enhancing the hydrophilicity of the surface. This leads to the formation of a thicker hydrophilic layer, which prevents aggregation and results in more stable and smaller polymeric micelles.^71^ In general, a higher hydrophilic-to-hydrophobic block ratio of the block copolymers leads to an increase in the CVC and a decrease in the number of aggregations, resulting in the formation of smaller polymeric micelles.^65^ Therefore, the larger diameter of the DM2 polymeric micelles compared to the DM4 polymeric micelles is likely due to the shorter PEG chain length, which resulted in a lower hydrophilic-to-hydrophobic block ratio in the DM2 structure. On the other hand, when the PEG length of the copolymer was constant, the mPEG_45_-*b*-PMEMA_91_ block copolymer with a linear MEMA segment formed a stable hydrophobic core more easily, resulting in a large size of M2 polymeric micelles. In comparison, the mPEG_45_-*b*-PDEA_105_ block copolymer with a DEA segment containing diethyl chains has greater difficulty stabilizing the hydrophobic core, leading to a smaller size of D2 polymeric micelles.

Hydrodynamic diameters of DM2, DM4, M2, and D2 polymeric micelles determined using DLS were 720 ± 260, 350 ± 140, 740 ± 290, and 120 ± 60 nm, respectively (Table 2). The trend of the hydrodynamic diameters of these polymeric micelles was similar to their diameters characterized using TEM. It was also noticed that the DM4 and D2 polymeric micelles exhibited a significant increase in size in DLS measurements compared to TEM measurements. The large hydrodynamic size of the DM4 polymeric micelle determined by DLS can be attributed to its high hydrophilic/hydrophobic block ratio of 0.85, which promoted the formation of a hydration shell around the polymeric micelles. This hydration layer resulted in larger size measurements than TEM, which measures the core diameter of the dried polymeric micelles. Regarding segment hydrophilicity, DEA is generally considered more hydrophilic than MEMA due to its tertiary amine group that can form ionic interactions with water. Therefore, the D2 polymeric micelle showed a more pronounced size increase in the DLS measurements.

The zeta potentials of DM2, DM4, M2, and D2 polymeric micelles were 26.3 ± 0.7, 28.0 ± 0.5, −42.4 ± 0.2, and 24.5 ± 0.9 mV, respectively (Table 2). The presence of tertiary amine groups on the DEA segments allowed them to be protonated at both neutral and acidic pH, contributing to the positive charge. In contrast, the methoxy (−OCH_3_) group and an ethoxy chain of MEMA are both polar and capable of forming hydrogen bonds with water, making the M2 polymeric micelles present a negative charge. Besides, the charge shielding effects of PEG can reduce the zeta potential of the polymeric micelles,^72^ which may explain why there is no notable difference between the zeta potentials of DM2 and DM4 polymeric micelles.

The stimuli responsiveness of polymeric micelles was also revealed using TEM. The size of the polymeric micelles did not show a significant change upon irradiation with ultrasound (1 MHz, 0.5 W/cm^2^) under the TEM images (Figure 2c). Typically, after ultrasound irradiation, numerous bubbles form, expand, and burst rapidly, with the energy released during these bursts causing the polymeric micelles to rupture. However, the subsequent rearrangement resulted in no significant change in the size of polymeric micelles. On the other hand, the destruction of the structures of DM2, DM4, and D2 polymeric micelles was observed in the TEM images in the acidic environment of pH 5.5, while the M2 polymeric micelle exhibited structural stability (Figure 2d). The dissociation of DM2 polymeric micelle at low pH was also observed in the cryo-TEM image (Figure S9). These results should be due to the protonation of the tertiary amine group of DEA to destabilize the structures of polymeric micelles.

**Figure 3:**
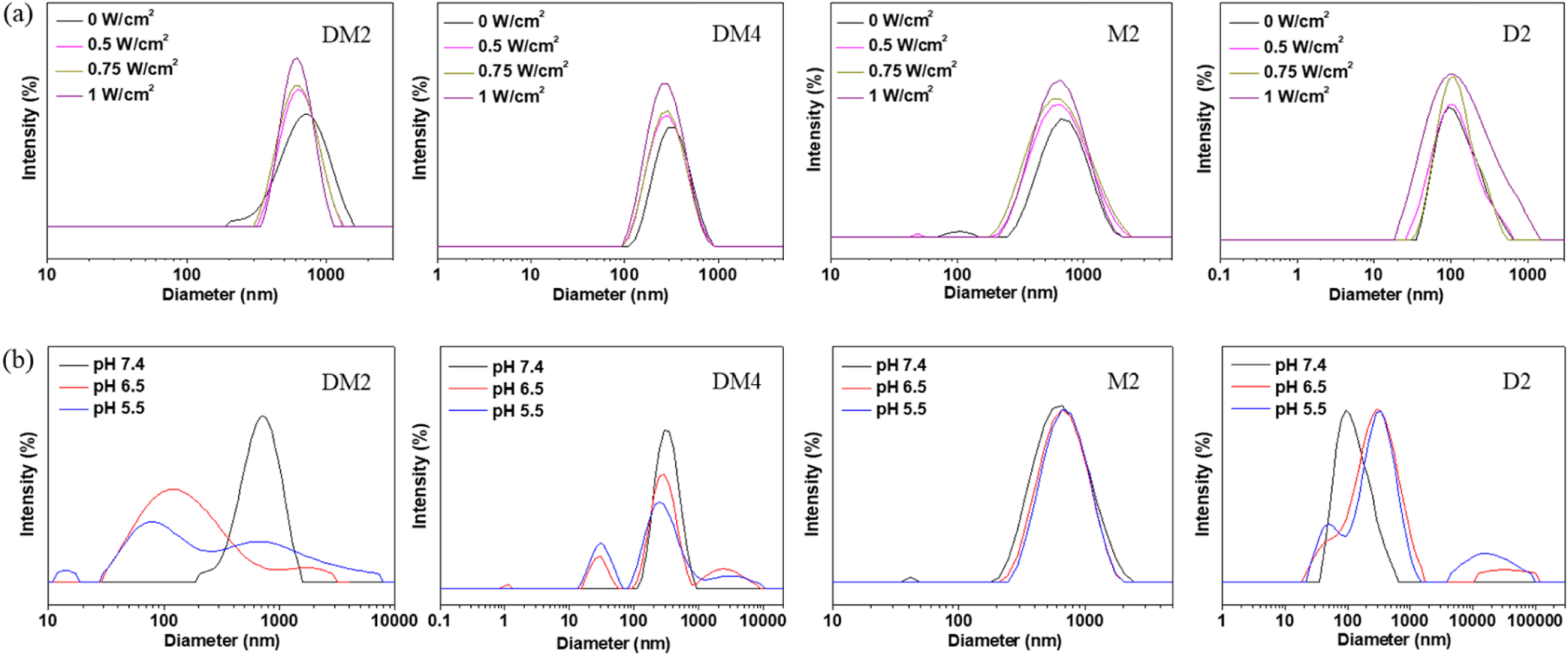
Hydrodynamic diameter changes of polymeric micelles (a) under ultrasound irradiation (1 MHz, pH 7.4) with different power densities and (b) at different pH values.

The effects of ultrasound and pH on the structural changes of polymeric micelles were further investigated by measuring the hydrodynamic diameter changes of polymeric micelles through DLS. After the ultrasound irradiation (1 MHz, 0.5 W/cm^2^), the hydrodynamic diameters of DM2, DM4, and M2 polymeric micelles were decreased to ∼630, 300, and 690 nm, respectively, while the D2 polymeric micelle exhibited no significant changes (Figure 3a). It was also noticed that the hydrodynamic diameter of DM2, DM4, and M2 polymeric micelles were further decreased with increasing ultrasound power density. These results indicated that polymeric micelles containing MEMA segments were sensitive to ultrasound irradiation, as evidenced by a decrease in their hydrodynamic diameters. However, the extent of diameter reduction was not significant due to the rapid rearrangement of the block copolymers once the ultrasound was removed.

On the other hand, the hydrodynamic diameters of DM2, DM4, and D2 polymeric micelles were decreased to ∼80, 30, and 50 nm at pH 5.5, respectively, while the M2 polymeric micelle exhibited structural stability (Figure 3b). These pH-induced hydrodynamic diameter changes of polymeric micelles were consistent with the observations under TEM images (Figure 2d), where the protonation of the tertiary amine groups within DEA led to structural disruption of DM2, DM4, and D2 polymeric micelles. The stability of the DM2 and DM4 polymeric micelles was also studied by placing polymeric micelles in PBS solutions for 2 days. The results showed that the hydrodynamic diameter distribution of DM2 and DM4 polymeric micelles remained unchanged (Figure S10), indicating that the stability of these polymeric micelles supports their potential applications in biomedicine.

### 3.5 Drug Encapsulation and Drug Loading Efficiency of Polymeric Micelles

The drug loading efficiency (DLE) and drug loading content (DLC) of polymeric micelles were further investigated by encapsulating DOX into the polymeric micelles, where DOX would be presented inside the hydrophobic core of polymeric micelles (Scheme 1b). A characteristic peak of DOX was observed at 480 nm in the absorption spectra of DOX-loaded polymeric micelles (Figure S11). Thus, the absorption peak of pure DOX·HCl can be obtained by subtracting the absorption of pure polymeric micelles from DOX-loaded polymeric micelles. A calibration curve was constructed using different concentrations of DOX solution to calculate DOX amount in the polymeric micelles (Figure S12). The DLE of DM2, DM4, M2, and D2 polymeric micelles were 32.1, 29.5, 31.4, and 27.4 wt %, respectively (Table S2). The DLC of DM2, DM4, M2, and D2 polymeric micelles were 8.0, 7.4, 7.9, and 6.9 wt %, respectively. DLE is typically influenced by factors such as drug-polymer compatibility (e.g., hydrogen bonding, hydrophobic interactions, electrostatic interactions, and chemical cross-linking), size, and block copolymer length.^73−76^ In this study, the DM2 polymeric micelles exhibited higher DLE and DLC than the DM4 polymeric micelle, which could be attributed to the larger diameter and higher hydrophobic portion of the DM2 polymeric micelle compared to the DM4 polymeric micelle. Specifically, the hydrophobic interactions between the hydrophobic DOX drug and the hydrophobic tail of the DM2 polymer led to an increase in drug loading efficiency.^76^

### 3.6 *In Vitro* Drug Release Experiment of Polymeric Micelles

**Figure 4:**
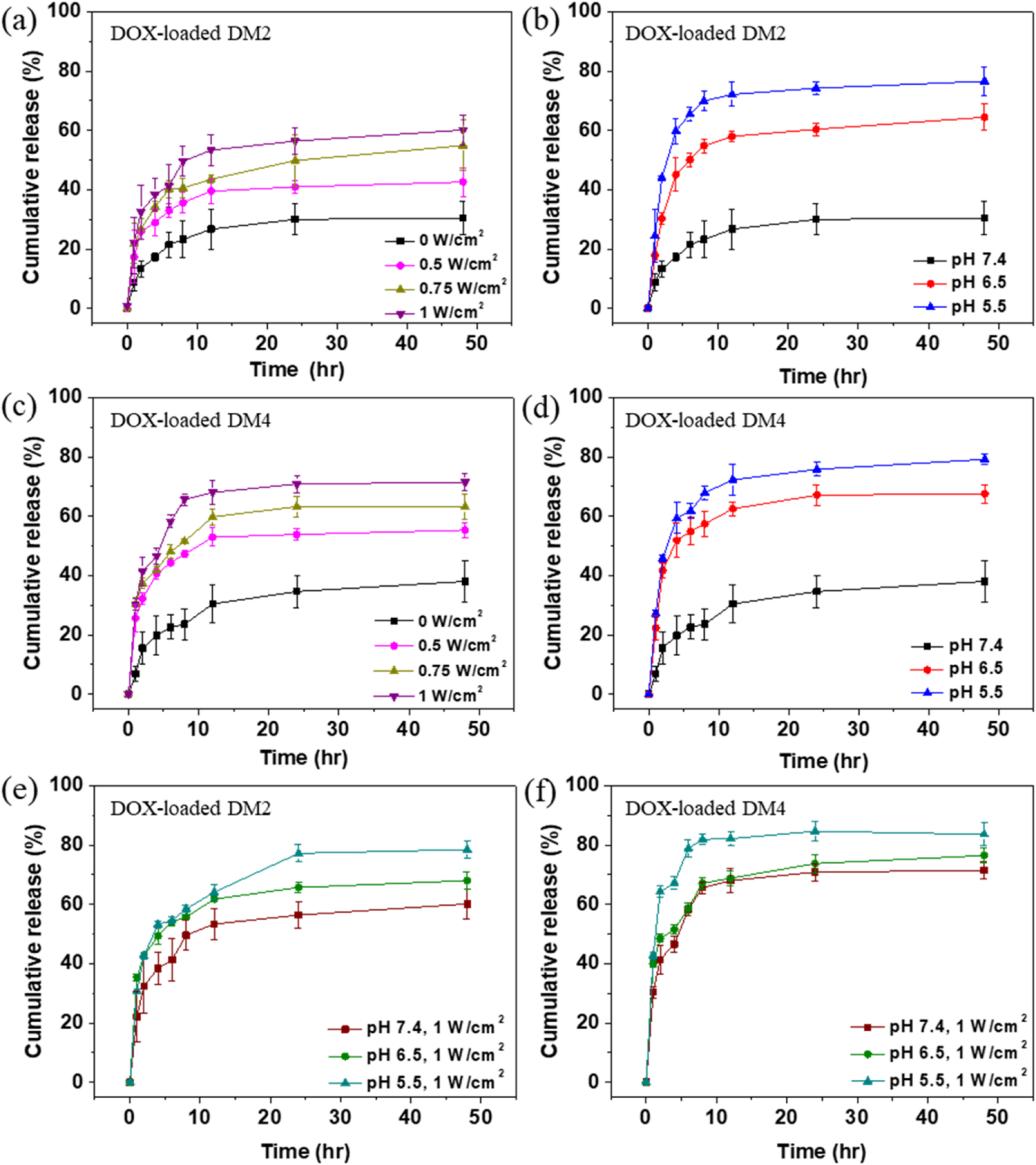
*In vitro* drug release of polymeric micelles at different time points. (a) DOX-loaded DM2 at pH 7.4 with different ultrasound power densities. (b) DOX-loaded DM2 at different pH values without ultrasound irradiation. (c) DOX-loaded DM4 at pH 7.4 with different ultrasound power densities. (d) DOX-loaded DM4 at different pH values without ultrasound irradiation. (e) DOX-loaded DM2 under different pH values with the ultrasound power density of 1 W/cm^2^. (f) DOX-loaded DM4 under different pH values with the ultrasound power density of 1 W/cm^2^.

*In vitro* drug release tests of DOX-loaded polymeric micelles were conducted by varying the power density of the ultrasound irradiation and the pH values of the solutions. The cumulative release rate of the DOX-loaded DM2 polymeric micelles without irradiation was approximately 30% over 48 h at pH 7.4. After ultrasound irradiation at power densities of 0.5, 0.75, and 1 W/cm^2^, the cumulative release rates of DOX-loaded DM2 polymeric micelles were 43%, 55%, and 60%, respectively (Figures 4a and S13a–d). When the pH of the solution was adjusted to 6.5 and 5.5, the cumulative release rates of the DOX-loaded DM2 polymeric micelles increased to 64% and 77%, respectively (Figures 4b and S13e,f). On the other hand, the cumulative release rate of DOX-loaded DM4 polymeric micelles was approximately 38% over 48 h at pH 7.4. Under ultrasound irradiation at 0.5, 0.75, and 1 W/cm^2^, the cumulative release rates of DOX-loaded DM4 polymeric micelles were 55%, 63%, and 71%, respectively (Figure 4c and S14a–d). At pH 6.5 and 5.5, the cumulative release rates of DOX-loaded DM4 polymeric micelles increased to 68% and 79%, respectively (Figures 4d and S14e,f). These results demonstrated the ultrasound- and pH-responsiveness of DM2 and DM4 polymeric micelles by performing the controlled drug release. Furthermore, the release profiles indicate that acidic pH had a more significant impact in facilitating drug release compared to the ultrasound irradiation used in this study. According to the morphological results shown in the TEM images and DLS analysis, this is likely because the low pH environment can cause permanent structural destruction of the polymeric micelles, whereas ultrasound only induces temporary structural changes in the polymeric micelles. Besides, the cumulative release rate of DOX-loaded DM2 polymeric micelles was slightly lower than that of DOX-loaded DM4 polymeric micelles. The DM2 polymeric micelle with a high proportion of hydrophobic segments performed the reduced drug release, which might be due to its more stable assembled structure resulting from the stronger intermolecular interactions. In addition, drugs required more time to diffuse from the larger diameter of DM2 polymeric micelles into the external solution.

The dual-responsive drug release experiment involving ultrasound- and pH-responsiveness was further performed. It was observed that DOX-loaded DM2 polymeric micelles exhibited a cumulative release rate of approximately 60%, 68%, and 78% over 48 h at pH 7.4, 6.5, and 5.5, respectively, after being exposed to ultrasound (1 MHz, 1 W/cm^2^) (Figures 4e and S15). Similarly, DOX-loaded DM4 polymeric micelles showed a cumulative release rate of approximately 71%, 76%, and 84% over 48 h at pH 7.4, 6.5, and 5.5, respectively, after ultrasound irradiation (1 MHz, 1 W/cm^2^) (Figures 4f and S16). These results demonstrated that dual stimulation with both ultrasound and pH led to cumulative drug release rates of 78% for DM2 and 84% for DM4 polymeric micelles over 48 h. These rates were higher than those achieved with ultrasound irradiation alone and slightly higher than those achieved with decreased pH alone.

The drug release profiles under various conditions were analyzed using the Korsmeyer-Peppas model to assess the release kinetics. The model follows the equation
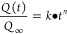
where *Q*(*t*) represents the cumulative drug release at time *t*, *Q*_∞_ is the theoretical maximum drug release, *k* is the kinetic constant that characterizes the release properties of the system, and *n* is the release exponent, which provides insights into the mechanism of drug release. An exponent *n* ≤ 0.5 suggests a Fickian diffusion mechanism, indicating that the release is governed by diffusion through the polymer matrix. When 0.5 < *n* < 1, the release follows anomalous transport involving both diffusion and polymer relaxation. An *n* = 1 indicates Case II transport, which correlates with zero-order kinetics, often linked to the relaxational release of the drug.^77−80^ By linearizing this equation through a log–log plot of log(*Q*(*t*)/*Q*_∞_) versus log(*t*), the parameters *k* and *n* were estimated (Figure S17 and Table S3). The early stage release kinetics, including potential burst release, were accounted for by excluding the initial *t* = 0 point from the fitting. This model enabled us to characterize controlled and sustained release behavior under different conditions, highlighting how the ultrasound power density and pH influence drug release rates from the polymeric micelles. From the data, all the values for the release exponent n were lower than 0.5, indicating that the drug release followed a Fickian diffusion mechanism, where the release is primarily driven by simple diffusion through the polymer matrix. Overall, these results showed that the primary driving force for drug release from the polymeric micelles is the permanent structural destruction induced by the low pH environment.

As controls, the drug release experiments were also conducted with DOX-loaded M2 and DOX-loaded D2 polymeric micelles. The results showed that the release profiles of the DOX-loaded M2 polymeric micelles were affected by ultrasound irradiation, showing their cumulative release rates were changed from 38% to 64% after ultrasound irradiation at a power density of 1 W/cm^2^, while the pH values of solution did not significantly affect the drug release manner (Figures S18 and S20a,b). Conversely, the release profiles of the DOX-loaded D2 polymeric micelles were more influenced by the pH values of solutions compared to the ultrasound effect, showing their cumulative release rates were changed from 35 to 84% after altering the pH from 7.4 to 5.5 (Figures S19 and S20c,d). These findings further demonstrated that the ultrasound- and pH-sensitive motifs in the polymeric micelle structure played a critical role in controlling drug release under different conditions.

### 3.7 *In Vitro* Cytocompatibility Study of Polymeric Micelles and DOX-Loaded Polymeric Micelles

**Figure 5:**
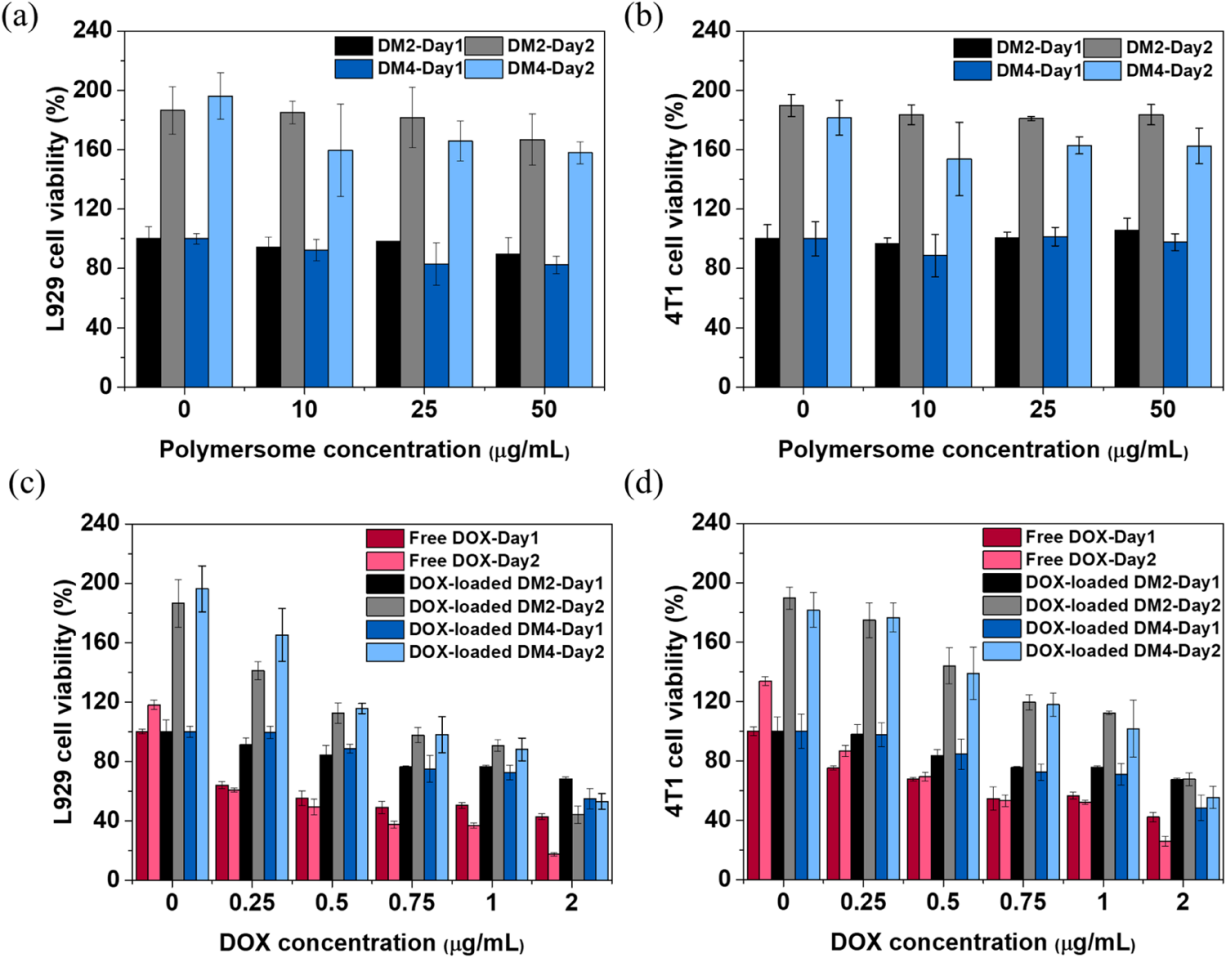
*In vitro* cytotoxicity of polymeric micelles. (a) L929 and (b) 4T1 cells were treated with polymeric micelles at varying concentrations. (c) L929 and (d) 4T1 cells were treated with DOX-loaded polymeric micelles at different concentrations, with the DOX concentrations subsequently calculated for plotting.

The cytocompatibility of polymeric micelles was evaluated using a CCK-8 assay. Different concentrations of polymeric micelles were cocultured with L929 normal cells (pH= ∼ 7.3) and 4T1 breast cancer cells (pH= ∼ 6.9). The results showed that the cell viability of both cell types remained above 80% on day 1, with no noticeable inhibition observed even on day 2. The sustained proliferation indicated that the polymeric micelles exhibited low cytotoxicity toward both cell types (Figure 5a,b).

To further evaluate the cytotoxicity of DOX-loaded polymeric micelles on L929 or 4T1 cells, solutions containing DOX-loaded polymeric micelles were diluted and cocultured with L929 or 4T1 cells (Figure 5c,d). On day 1, the cell viability of L929 cells remained above 60% even at the highest concentration of DOX-loaded DM2 polymeric micelles. However, significant inhibition was observed at a concentration of 2 μg/mL. The cytotoxic effect became more pronounced with increasing concentrations of DOX-loaded DM2 polymeric micelles, adversely affecting cell proliferation. Likewise, the viability of 4T1 cells declined to nearly 68% at a concentration of 2 μg/mL, with a trend of cytotoxicity similar to that of the L929 cells. When the medium containing DOX-loaded DM4 polymeric micelles was applied to both cells, no significant difference was observed compared to DOX-loaded DM2 polymeric micelles. After 2 days of culture, cell viability slightly increased at concentrations below 1 μg/mL, but cell proliferation decreased as the DOX concentration increased.

### 3.8 *In Vitro* Anticancer Effects of DOX-Loaded Polymeric Micelles under Ultrasound Irradiation

**Figure 6:**
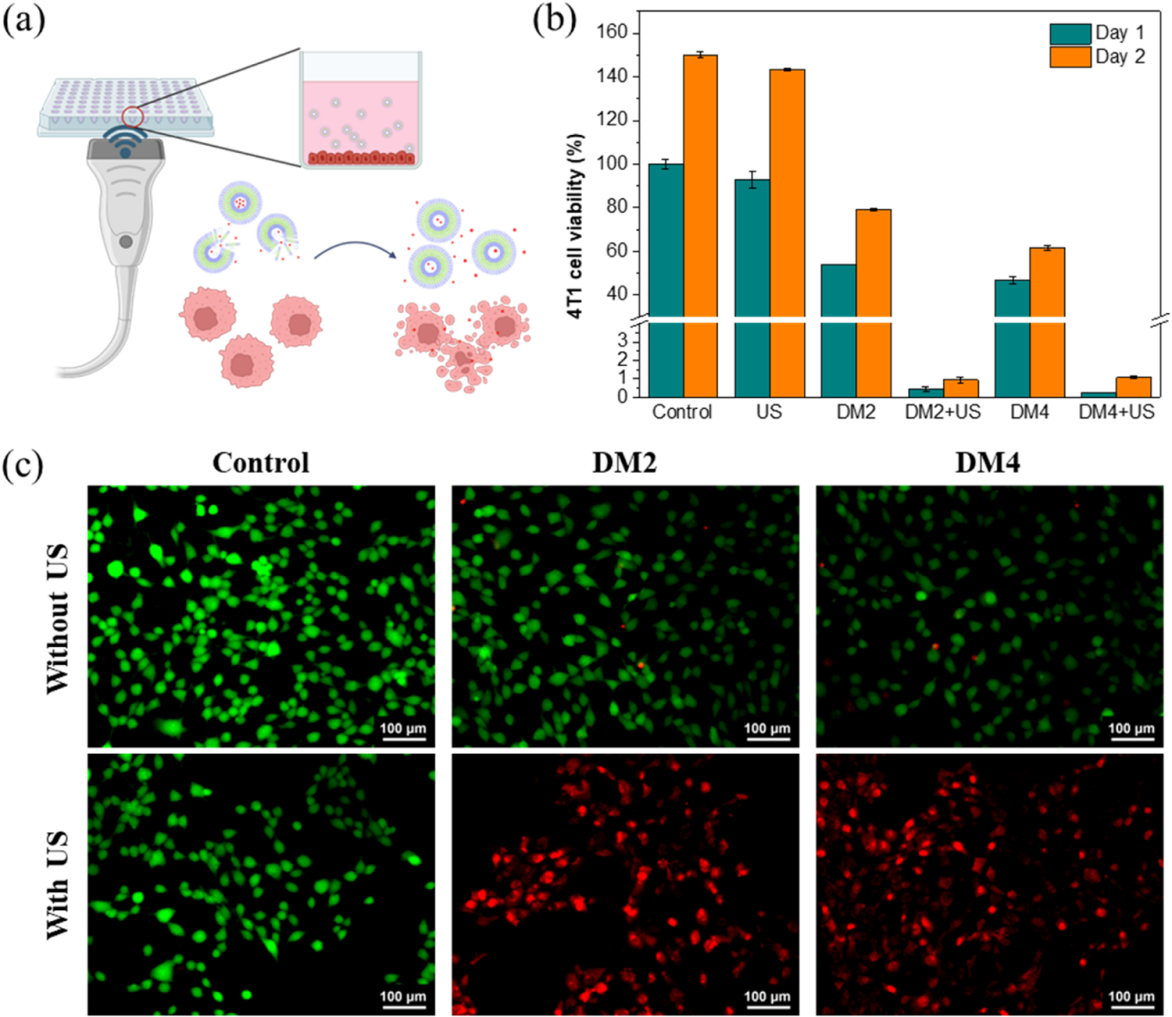
(a) Schematic illustration of the cells incubated with Dox-loaded polymeric micelles for ultrasound-triggered drug release. (b) CCK8 assay results and (c) live/dead staining images of 4T1 cells were used to evaluate the effect of Dox release from polymeric micelles for killing cancer cells.

To evaluate the anticancer efficacy of the DOX-loaded polymeric micelles, the CCK-8 assay was utilized to assess the cell viability of breast cancer cell 4T1 under ultrasound irradiation. 4T1 cells were cocultured with DOX-loaded polymeric micelles (0.5 μg/mL) for 2 h, followed by ultrasound irradiation (1 MHz, 1 W/cm^2^) for 3 min (Figure 6a). The results showed that the proliferation of 4T1 cells was not efficiently inhibited in the absence of the ultrasound after 24 h, with cell viability remaining at approximately 54% and 47% for DM2 and DM4, respectively (Figure 6b). However, a noticeable inhibition was observed upon ultrasound application. After 1 day, cell viability drastically decreased to ∼0.4% in the DOX-loaded DM2 group and ∼0.3% in the DOX-loaded DM4 group. Examining the DLE results, it was noticed that DM2 possessed a higher DOX content (the DLE of DM2 was 32% compared to 29.5% in DM4). However, DM2, with a higher ratio of hydrophobic segments, might possess a more stable structure due to stronger molecular interactions, resulting in a lower extent of drug release.^81^ This stability may lead to a slower release of DOX under nonirradiated conditions, leading to higher cell viability. In contrast, DM4 with a looser structure, might release DOX more easily without the application of ultrasound. Therefore, the viability of 4T1 cells was relatively higher in the DM2 group. Nonetheless, both groups without ultrasound irradiation exhibited relatively poor anticancer performance compared with the ultrasound-treated groups. When ultrasound was applied, the structure of the polymeric micelles, especially those containing the ultrasound-responsive MEMA, was disrupted, leading to a more efficient release of DOX. This resulted in a significant reduction in cell viability in both groups. Additionally, live and dead assay staining was performed on the cells, showing that most cells exhibited good cell viability without ultrasound irradiation (Figure 6c). However, following exposure to ultrasound, almost all cells treated with DOX-loaded polymeric micelles displayed signs of apoptosis. The live and dead staining results were consistent with the CCK8 assay results. These cellular studies demonstrated that both kinds of DOX-loaded polymeric micelles exhibited excellent performance in inhibiting cancer cell growth under ultrasound irradiation with great controllability.

## 4 Conclusions

In this study, RAFT polymerization was employed to synthesize a series of stimulus-responsive block copolymers, including dual-responsive (ultrasound and pH) mPEG_45_-*b*-P(DEA_26_-*co*-MEMA_49_) and mPEG_90_-*b*-P(DEA_5_-*co*-MEMA_26_), as well as ultrasound-responsive mPEG_45_-*b*-PMEMA_91_ and pH-responsive mPEG_45_-*b*-PDEA_105_ block copolymers. The CVC of DM2 was lower than that of DM4 due to the lower hydrophilic-to-hydrophobic block ratio in the DM2 structure. Additionally, the DM2 polymeric micelle, with its larger diameter and lower hydrophilic-to-hydrophobic block ratio, exhibited a more remarkable ability to encapsulate DOX than the DM4 polymeric micelle. *In vitro* drug release experiments showed that both ultrasound and pH can trigger DOX release from polymeric micelles, with the permanent structural destruction caused by the low pH environment being the key driving force to release drugs from the polymeric micelles. The cumulative release rate of DOX-loaded DM2 polymeric micelles was lower than that of DOX-loaded DM4 polymeric micelles, indicating that the stable assembled structure of the DM2 polymeric micelle acts as a controlling barrier for the release of DOX as well as the larger diameter of DM2 polymeric micelle required more time for the drug to diffuse into the external solution. DOX-loaded DM2 and DM4 polymeric micelles further demonstrated their capability to control DOX release for treating cancer cells under ultrasound irradiation. To sum up, dual-stimuli responsive polymeric micelles formed using block copolymers with different hydrophilic/hydrophobic blocks ratios have been compared regarding their structures and drug release performance, offering more profound insights into the structure–property relationship of polymeric micelles for controlled drug release applications. To offer a more detailed understanding of the structure-properties relationship of stimuli-responsive amphiphilic block copolymers in forming polymeric vehicles, this copolymer system could be advanced by preparing them with identical hydrophobic blocks but varying PEG lengths. This approach would provide more comparable structural parameters for investigating self-assembled structures and their functional properties.

## Supporting Information Available

Synthesis routes, ^1^H NMR, ESI-MS, and FT-IR spectra of molecules and block copolymers; GPC profiles of macromers and polymers; fluorescent spectra and CVC determinations for block copolymers; Cryo-TEM image of DM2 polymeric micelle at pH 5.5; hydrodynamic diameter analysis of polymeric micelles; UV–vis spectra of polymeric micelles in the absence and presence of DOX·HCl; UV–vis spectra of DOX-loaded polymeric micelles for *in vitro* drug release under varying pH values and ultrasound power densities at different time points (PDF)
