# Supplementary material for: Fabrication of pH- and Ultrasound-Responsive Polymeric Micelles: The Effect of Amphiphilic Block Copolymers with Different Hydrophilic/Hydrophobic Block Ratios for Self-Assembly and Controlled Drug Release
Source: Biomacromolecules. 2025 Mar 11;26(4):2116–30. doi: 10.1021/acs.biomac.4c01202 (PMC12004527; doi:10.1021/acs.biomac.4c01202)
Supplement: Supplementary file 1 — bm4c01202_si_001.pdf [file bm4c01202_si_001.pdf]

Supporting Information

Fabrication of pH- and ultrasound-responsive polymeric micelles: the effect of amphiphilic block copolymers with different hydrophilic/hydrophobic block ratios for self-assembly and controlled drug release

Hong-Xiang Wei[a,1], Ming-Hsin Liu[b,1], Tzu-Ying Wang[a], Meng-Hsiu Shih[b], Jiashing Yu[b*], and Yi-Cheun Yeh[a*]

[a] Institute of Polymer Science and Engineering, National Taiwan University, Taipei 10617, Taiwan

[b] Department of Chemical Engineering, National Taiwan University, Taipei 10617, Taiwan

*Corresponding authors:

Jiashing Yu, E-mail: jiayu@ntu.edu.tw; Yi-Cheun Yeh, E-mail: yicheun@ntu.edu.tw

[1] These authors contributed equally to this work.

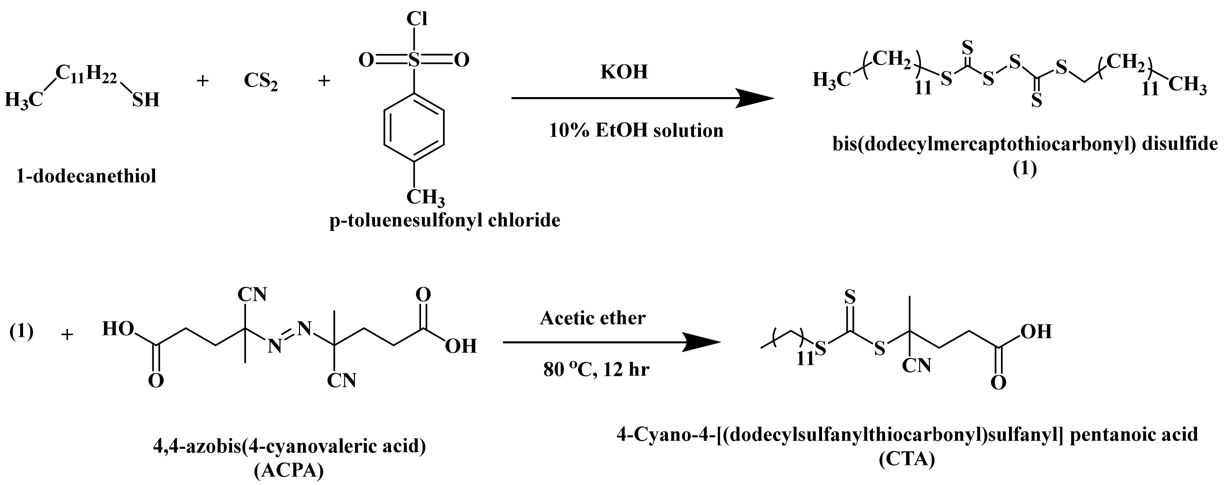

Scheme S1. Synthesis routes of 4-cyano-4-[(dodecylsulfanylthiocarbonyl)sulfanyl] pentanoic acid as a chain transfer agent (CTA) in the RAFT reaction.

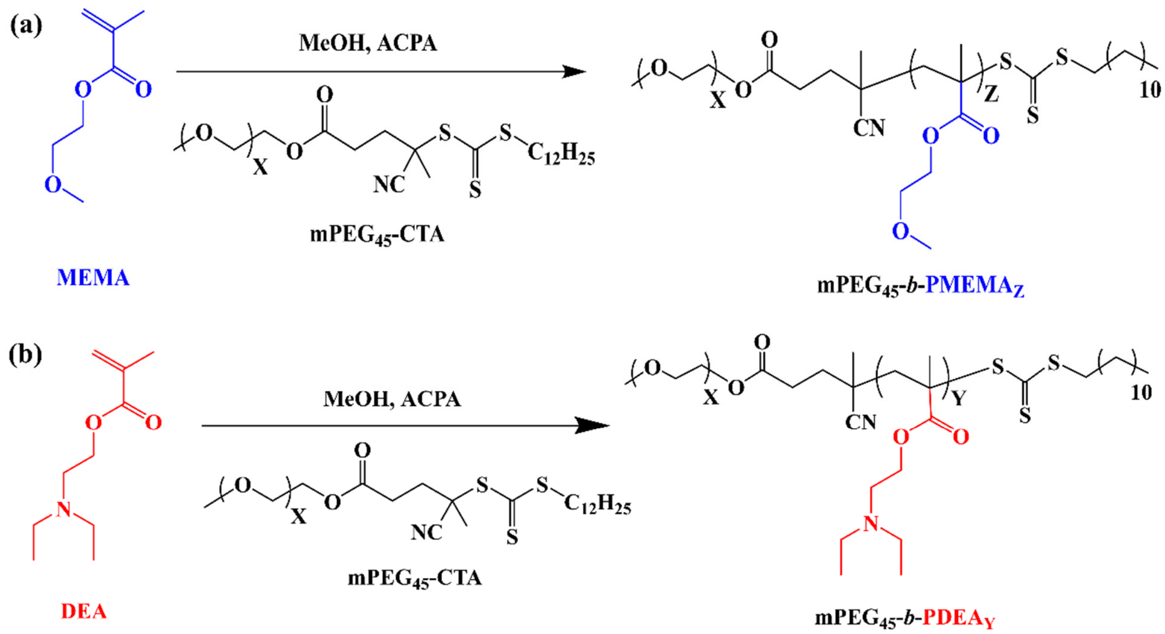

Scheme S2. Synthesis routes of (a) $mPEG_{45}$-*b*-$PMEMA_Z$ and (b) $mPEG_{45}$-*b*-$PDEA_Y$.

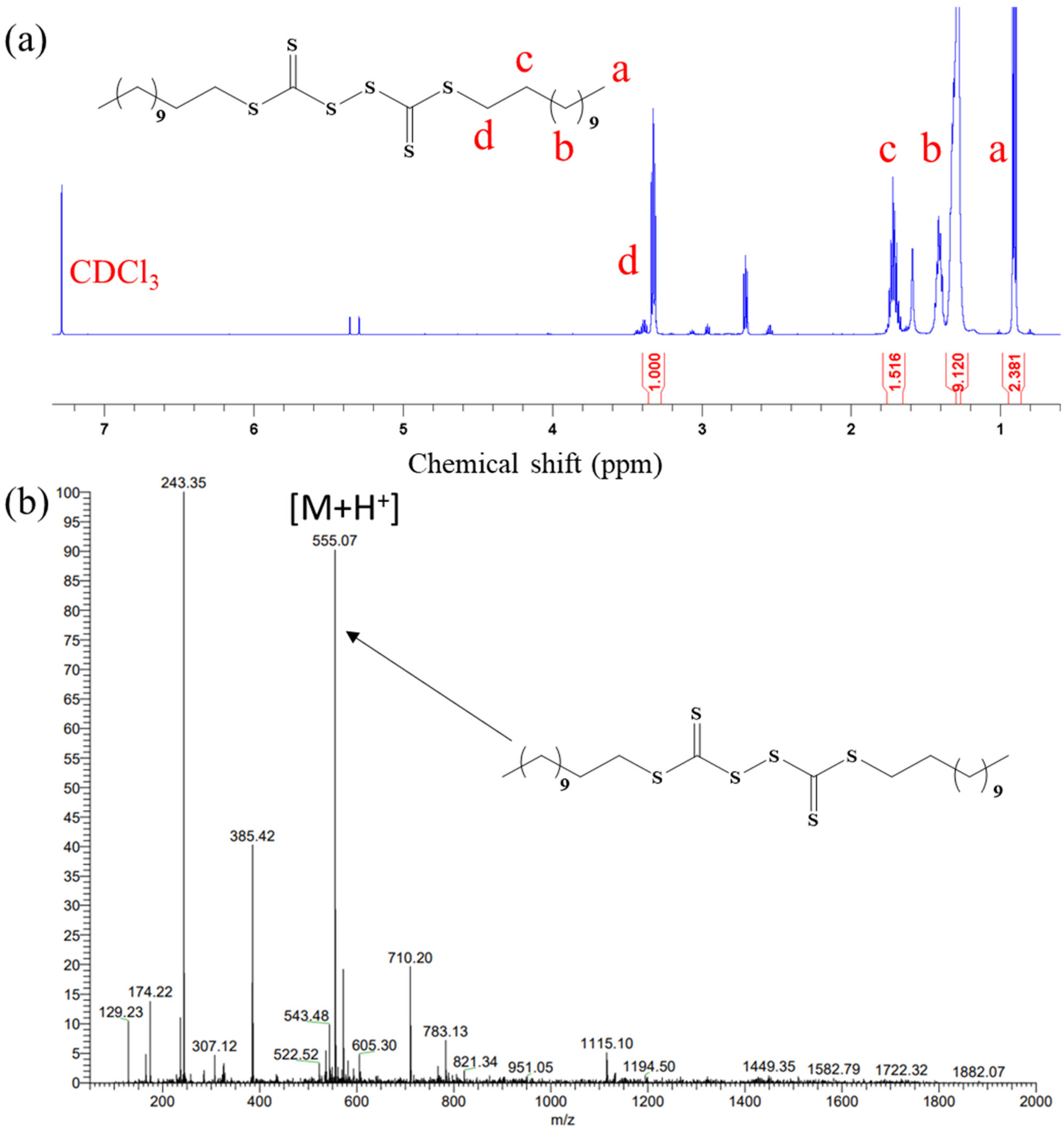

Figure S1. (a) ^{1}H NMR and (b) ESI-MS spectra of compound 1.

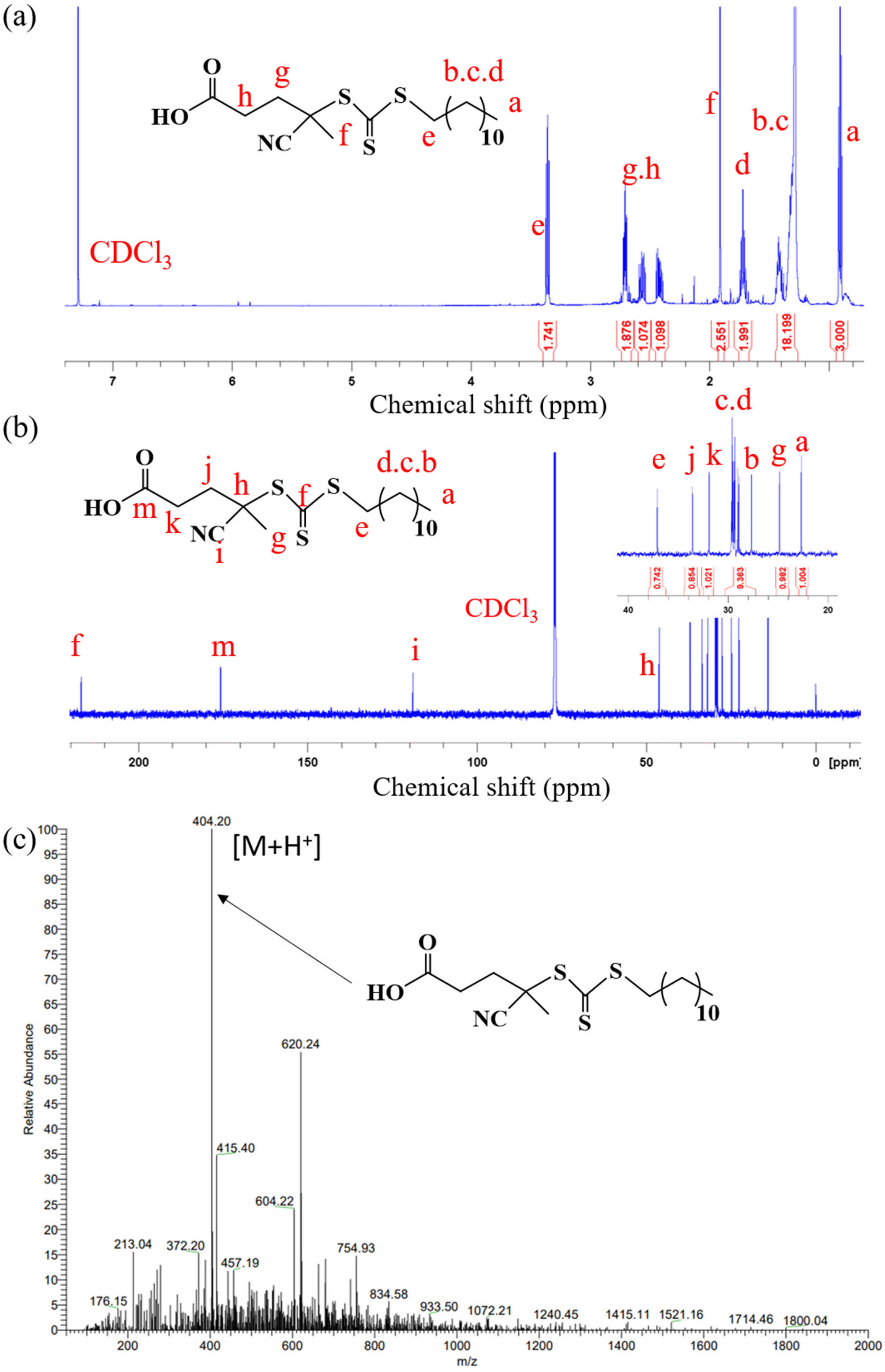

Figure S2. (a) ^{1}H NMR, (b) ^{13}C NMR, and (c) ESI-MS spectra of 4-cyano-4-[(dodecylsulfanylthiocarbonyl)sulfanyl] pentanoic acid (CTA).

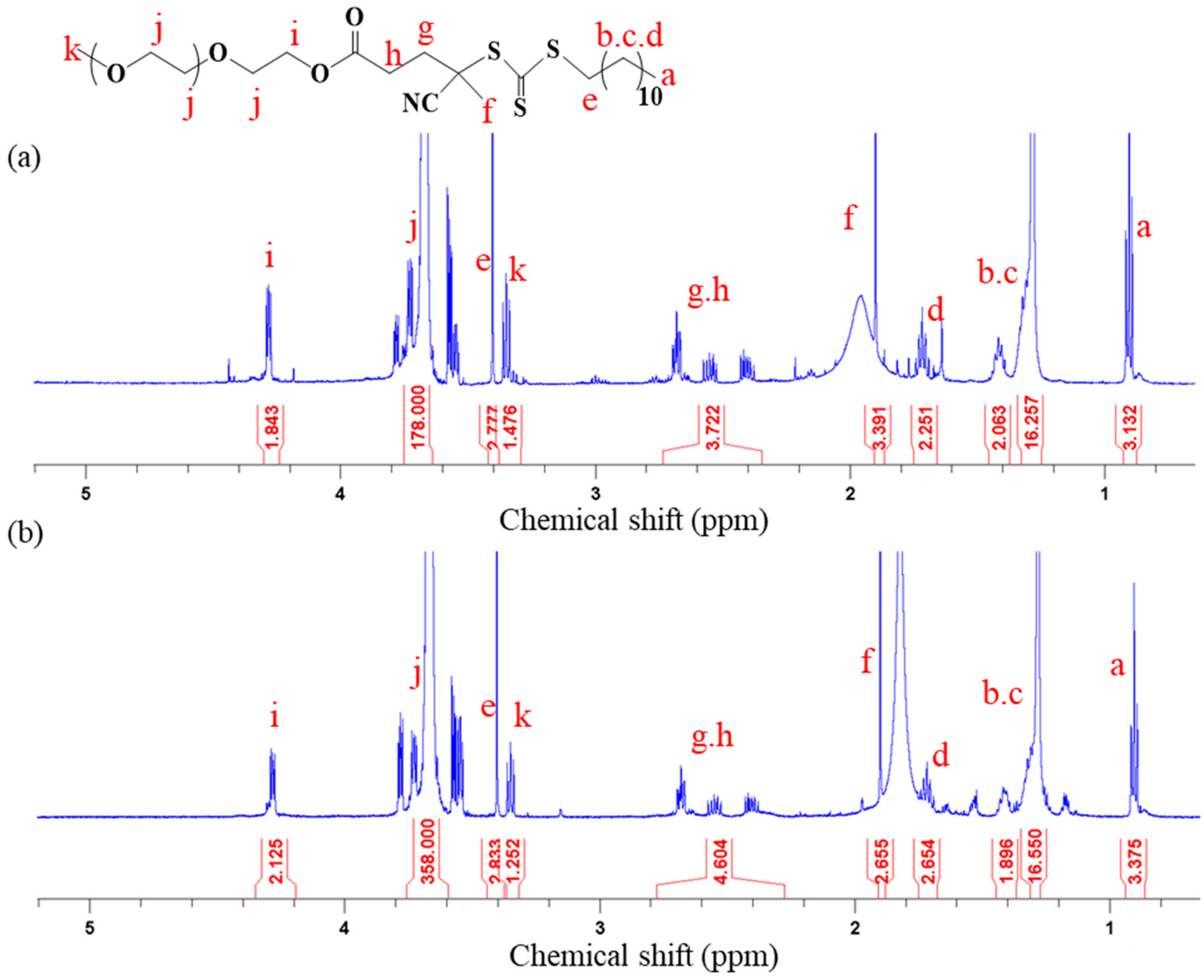

Figure S3. ^{1}H NMR spectra of (a) $mPEG_{45}$-CTA and (b) $mPEG_{90}$-CTA.

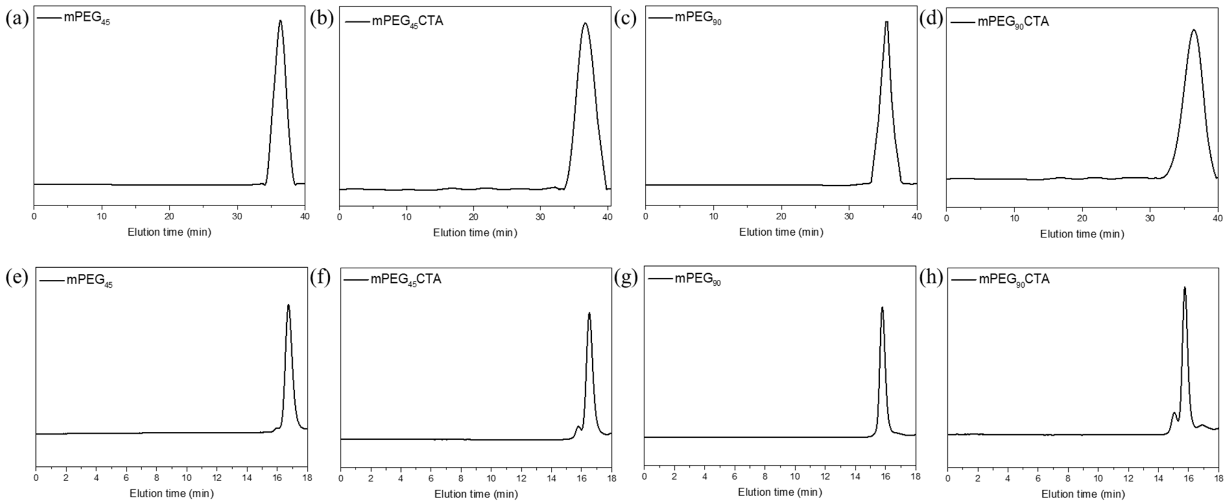

Figure S4. GPC traces of PEG and mPEG45-CTA samples by using (a-d) water and (e-h) THF as eluents.

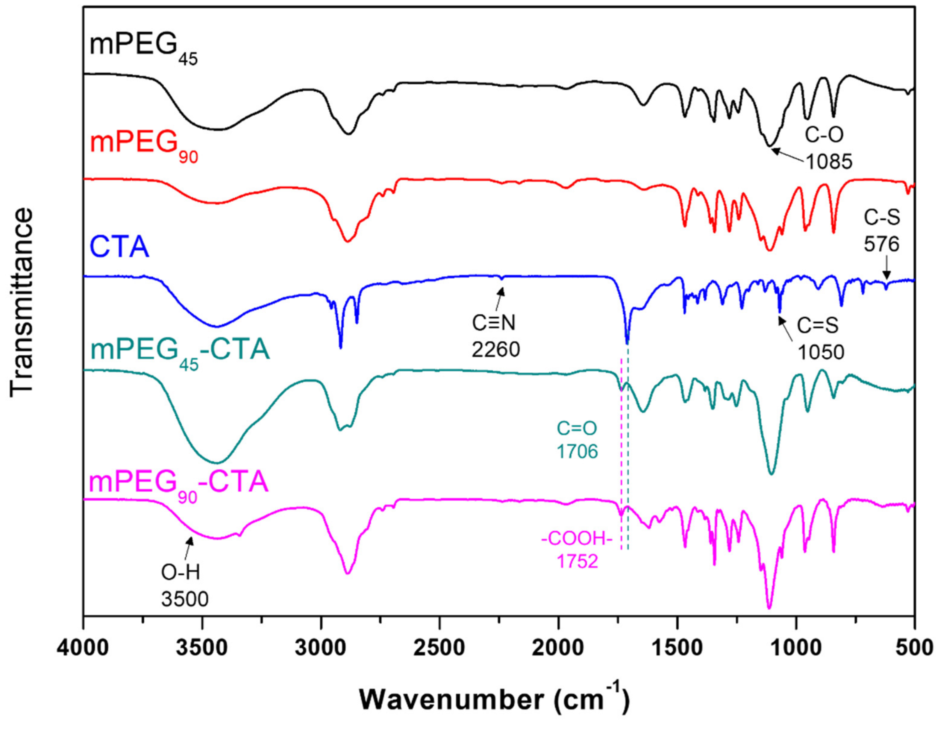

Figure S5. FTIR spectra of mPEG45, mPEG90, CTA, mPEG45-CTA, and mPEG90-CTA.

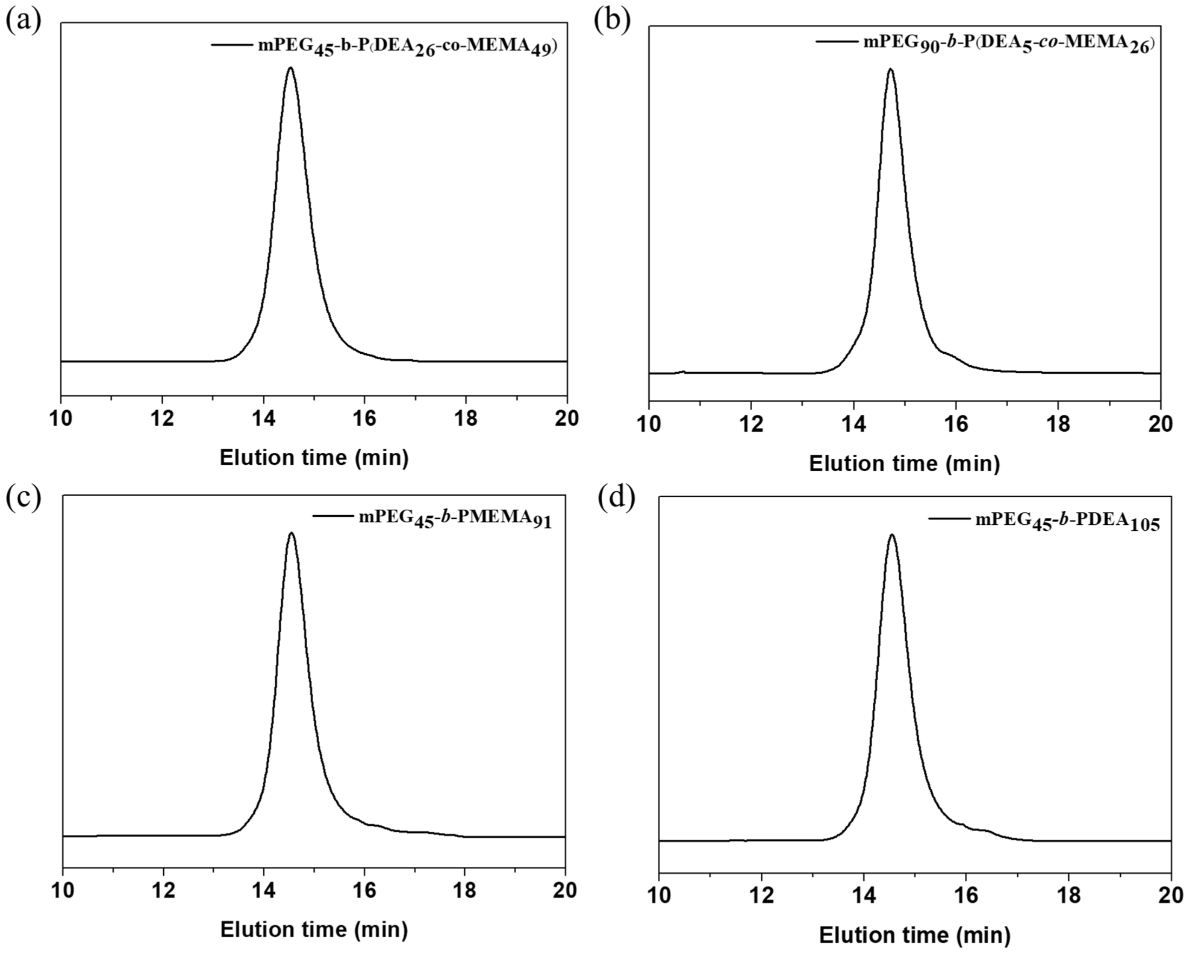

Figure S6. GPC traces of (a) mPEG$_{45}$-*b*-P(DEA$_{26}$-*co*-MEMA$_{49}$) (DM2), (b) mPEG$_{90}$-*b*-P(DEA$_{5}$-*co*-MEMA$_{26}$) (DM4), (c) mPEG$_{45}$-*b*-PMEMA$_{91}$ (M2), and (d) mPEG$_{45}$-*b*-PDEA$_{105}$ (D2) by using THF as eluent.

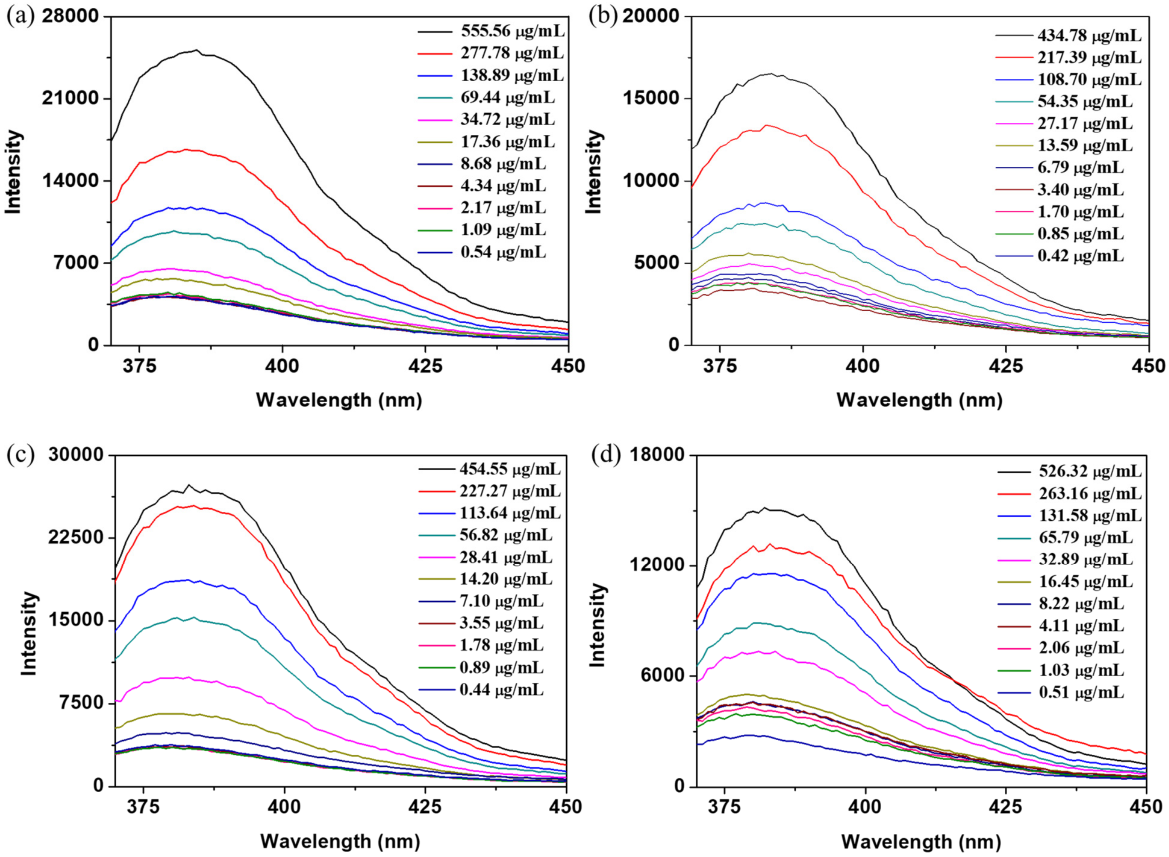

Figure S7. Fluorescence spectra of (a) DM2, (b) DM4, (c) M2, and (d) D2 block copolymers at different concentrations, with an excitation wavelength of 334 nm.

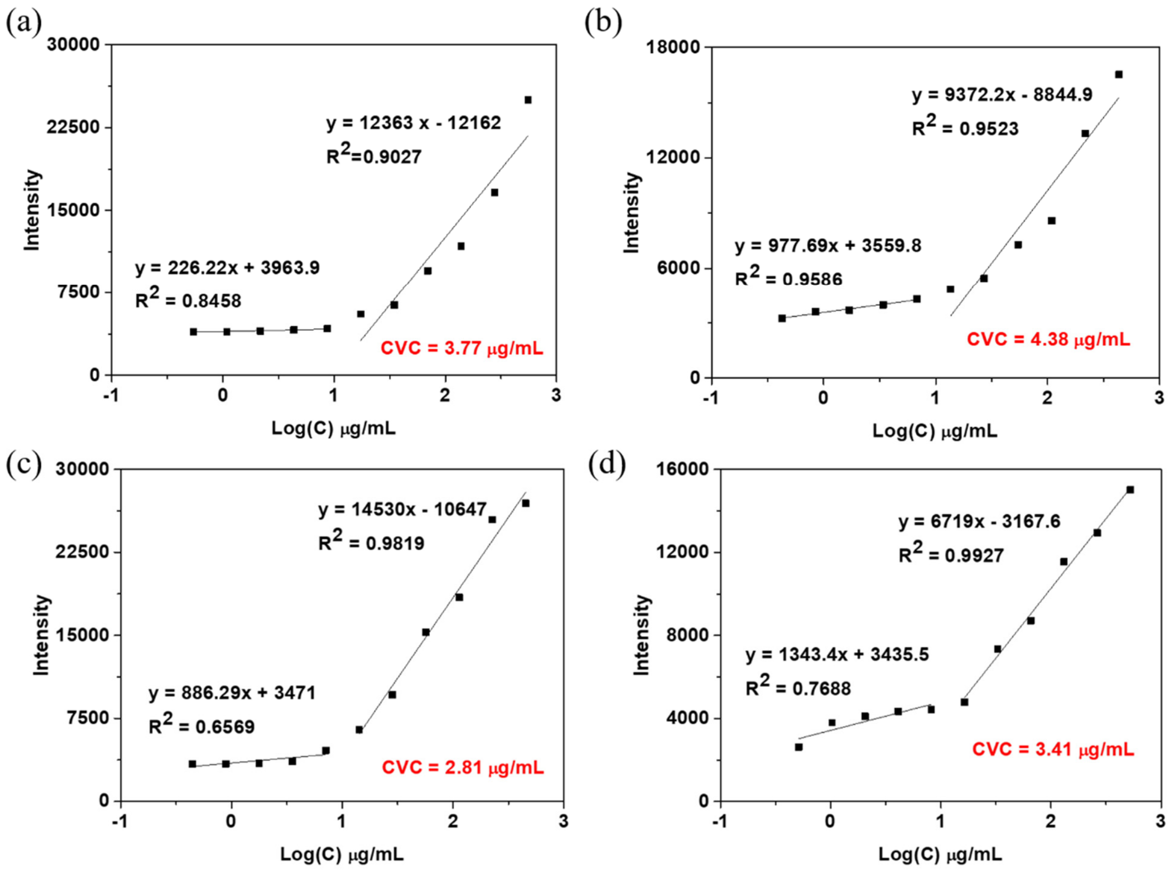

Figure S8. CVC determinations for (a) DM2, (b) DM4, (c) M2, and (d) D2 block copolymers.

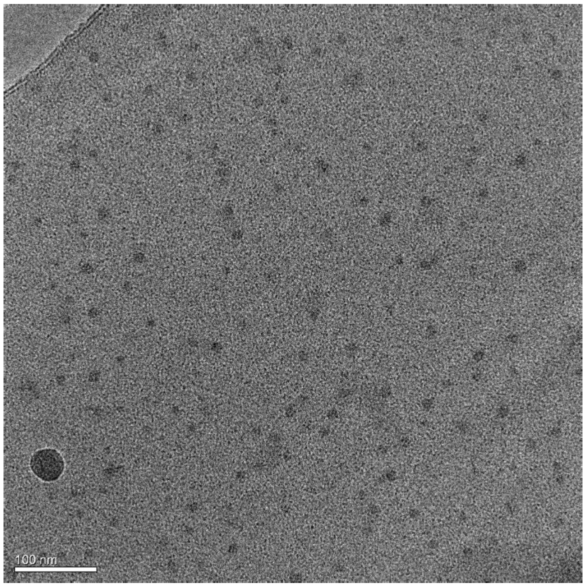

Figure S9. Cryo-TEM image of DM2 polymeric micelle at pH 5.5.

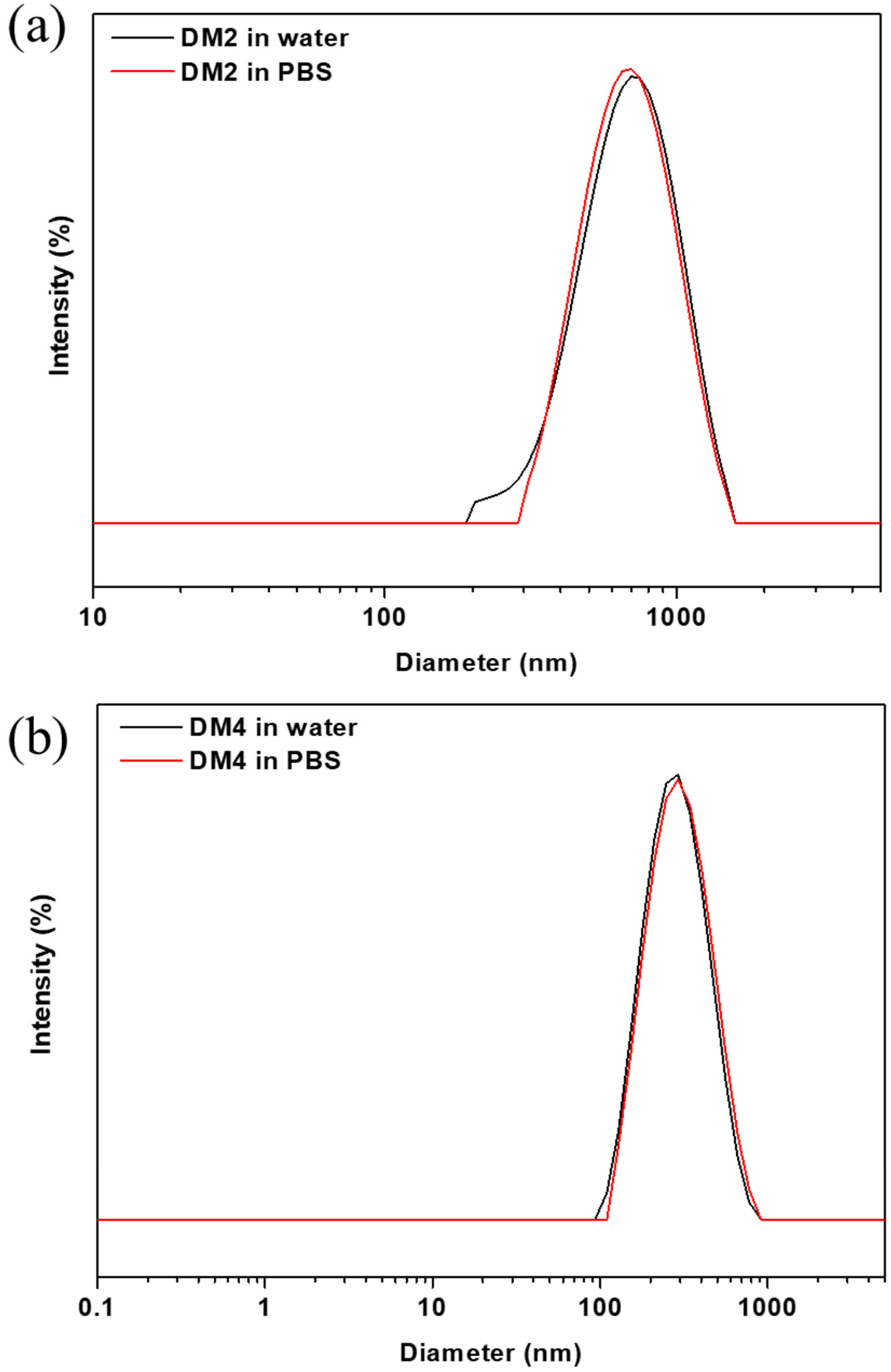

Figure S10. Hydrodynamic diameter analysis of (a) DM2 and (b) DM4 polymeric micelles in water and PBS solution after immersion for two days.

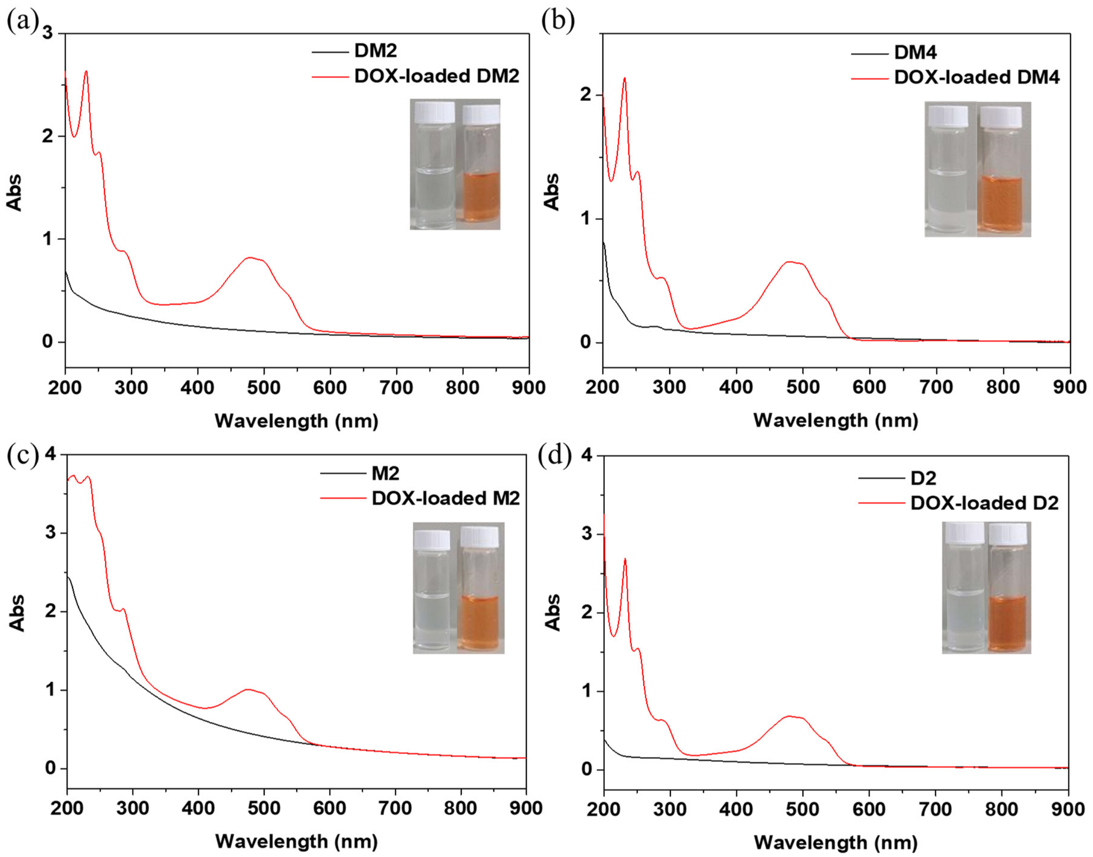

Figure S11. UV-vis spectra of polymeric micelles in the absence and presence of DOX·HCl. Inset: solution photographs of polymeric micelle (left) and DOX-loaded polymeric micelle (right).

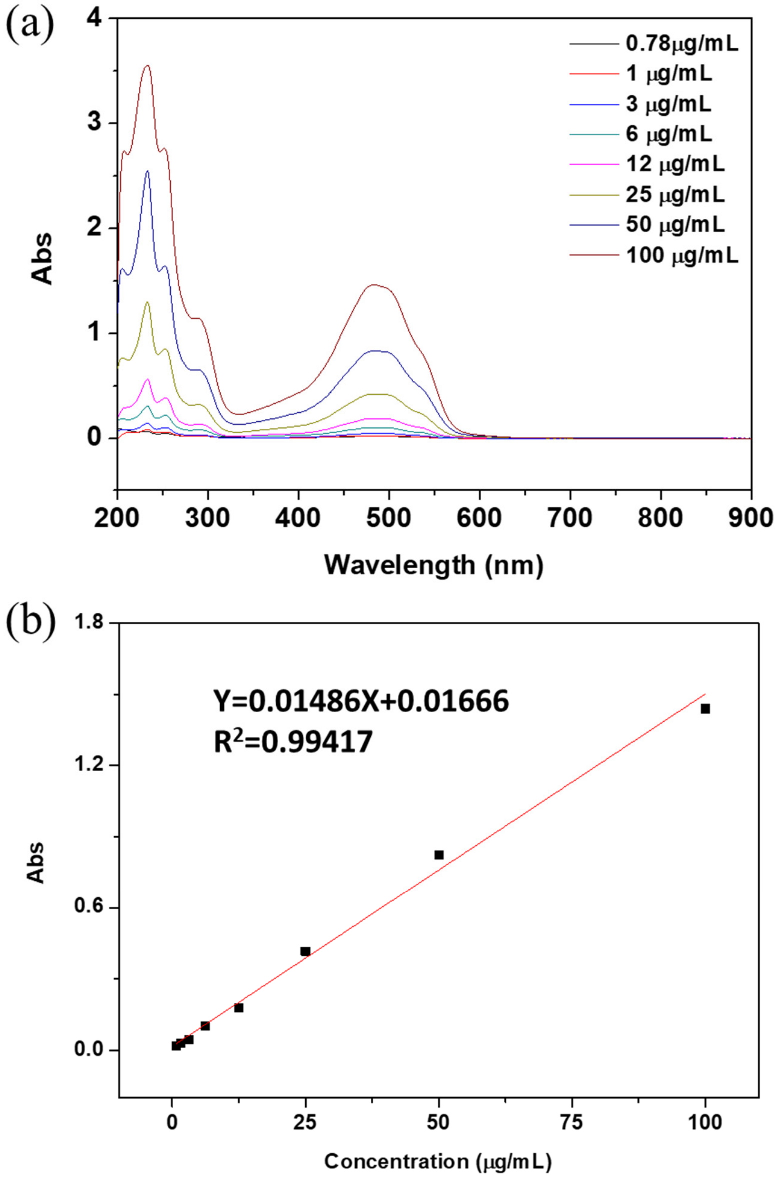

Figure S12. (a) UV-Vis spectra and (b) absorption calibration curve (wavelength= 480 nm) of different concentrations of DOX·HCl in PBS solution (pH 7.4).

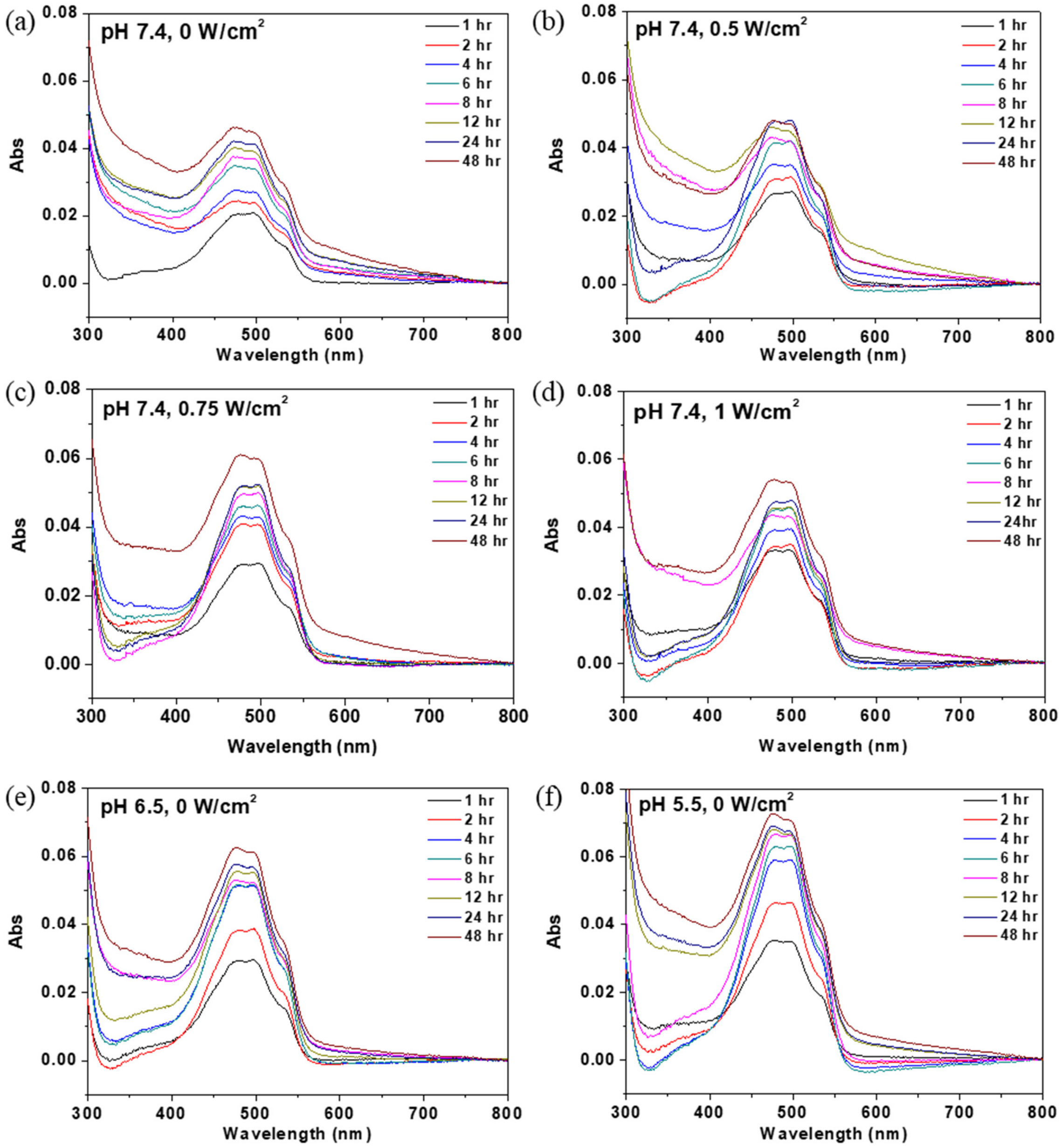

Figure S13. UV-Vis spectra of DOX-loaded DM2 polymeric micelle for *in vitro* drug release under varying pH values and ultrasound power densities at different time points.

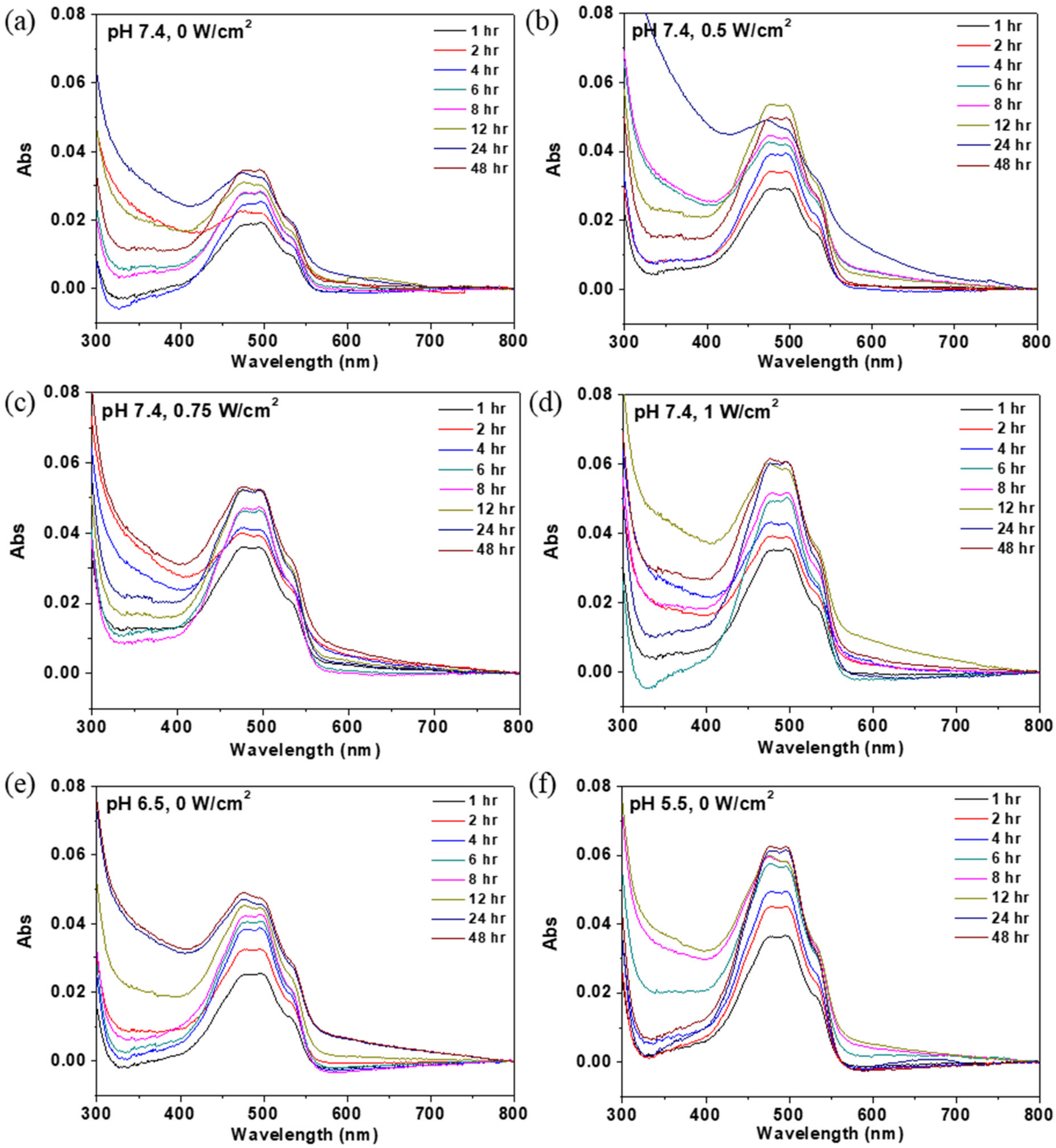

Figure S14. UV-Vis spectra of DOX-loaded DM4 polymeric micelle for *in vitro* drug release under varying pH values and ultrasound power densities at different time points.

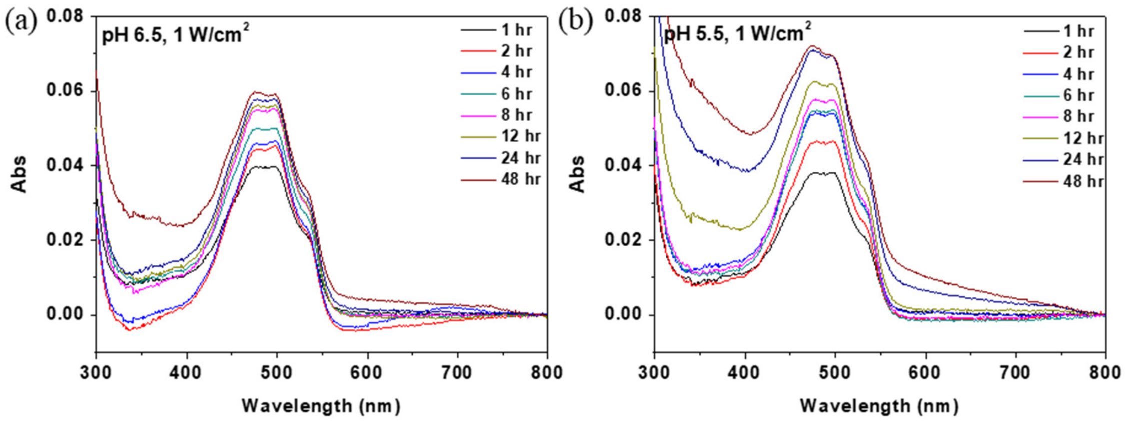

Figure S15. UV-Vis spectra of DOX-loaded DM2 polymeric micelle for *in vitro* drug release under varying pH values and ultrasound power densities at different time points.

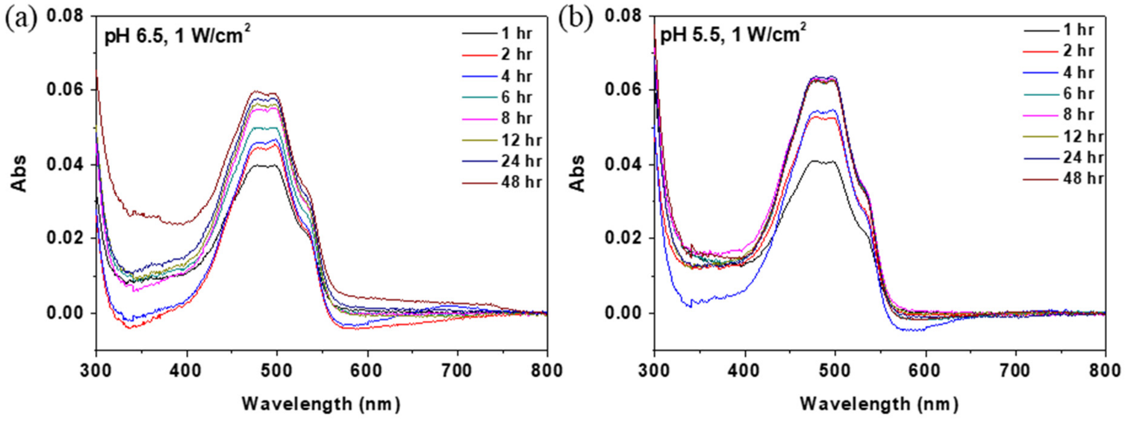

Figure S16. UV-Vis spectra of DOX-loaded DM4 polymeric micelle for *in vitro* drug release under varying pH values and ultrasound power densities at different time points.

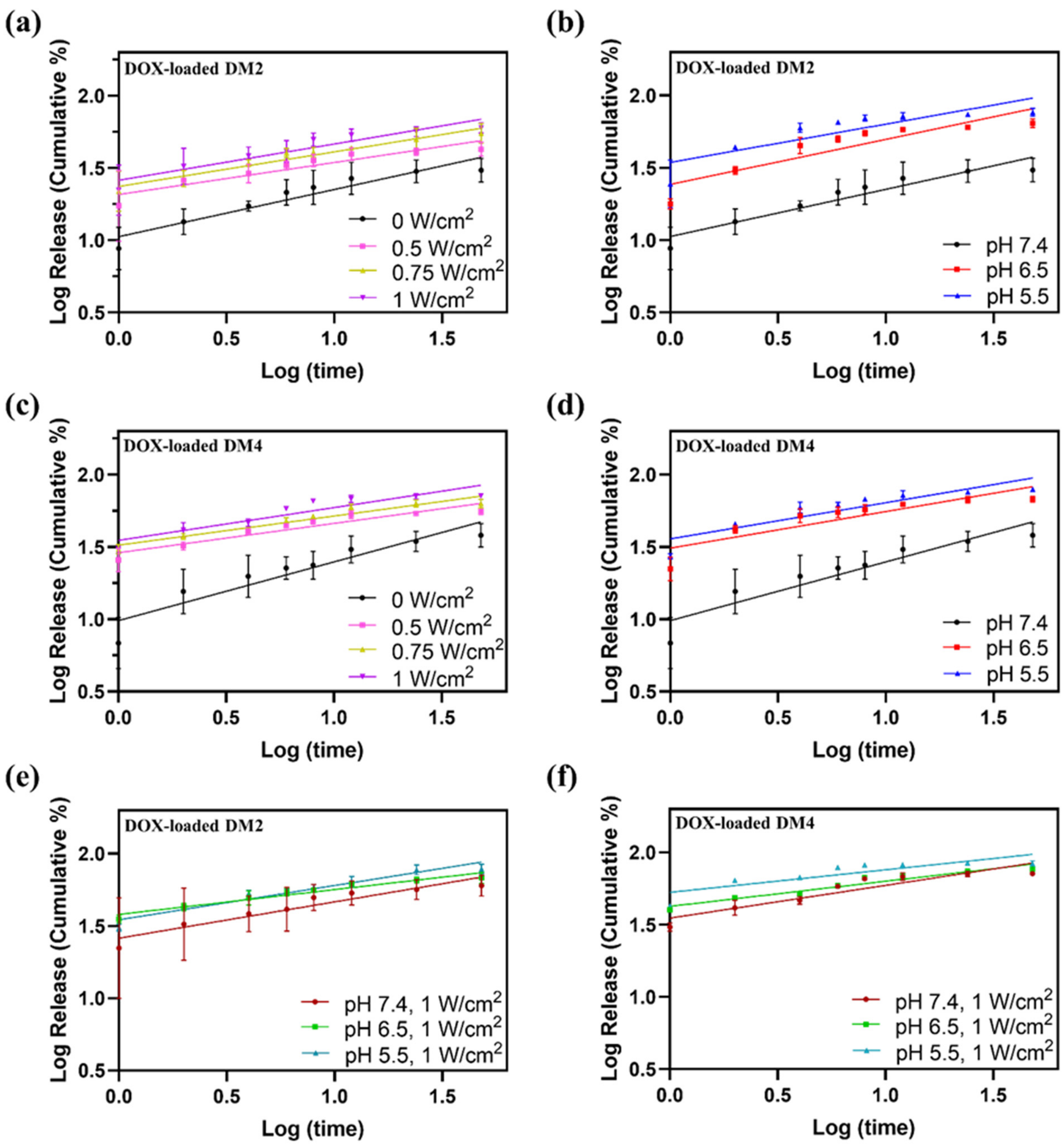

Figure S17. *In vitro* drug release of polymeric micelles fitted to the Korsmeyer-Peppas model $\frac{Q(t)}{Q\infty} = k \cdot t^n$ at different time points: (a) DOX-loaded DM2 polymeric micelle at pH 7.4 with different ultrasound power densities. (b) DOX-loaded DM2 polymeric micelle at different pH values without ultrasound irradiation. (c) DOX-loaded DM4 polymeric micelle at pH 7.4 with different ultrasound power densities. (d) DOX-loaded DM4 polymeric micelle at different pH values without ultrasound irradiation. (e) DOX-loaded DM2 polymeric micelle under different pH values with an ultrasound power density of 1 W/cm². (f) DOX-loaded DM4 polymeric micelle under different pH values with an ultrasound power density of 1 W/cm².

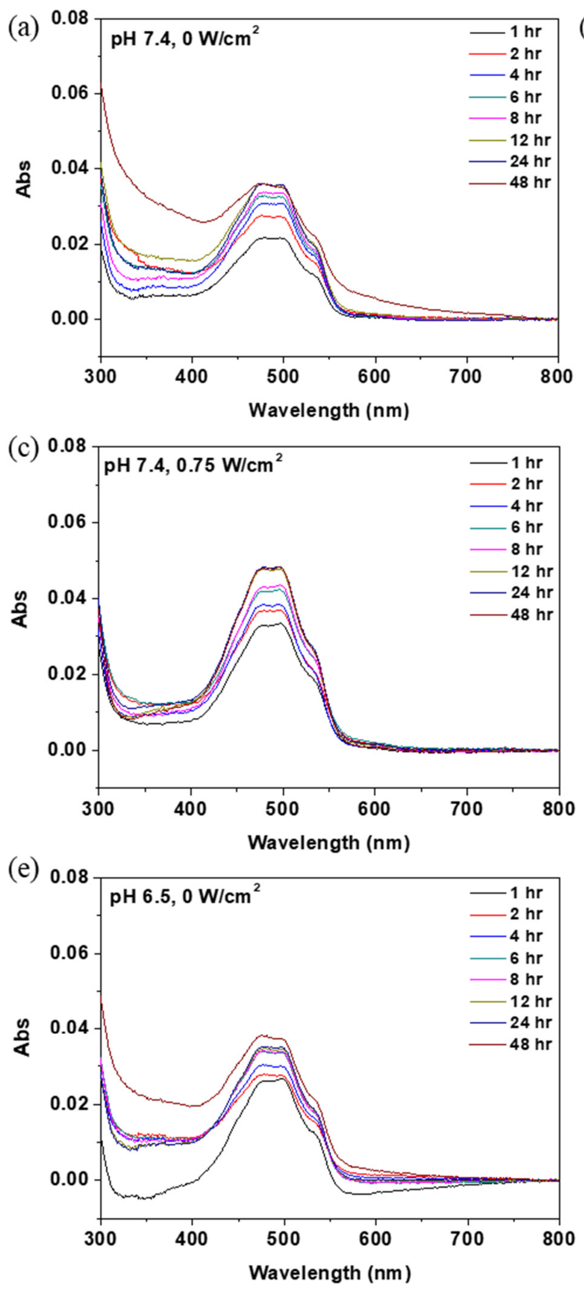

Figure S18. UV-Vis spectra of DOX-loaded M2 polymeric micelle for in vitro drug release under varying pH values and ultrasound power densities at different time points.

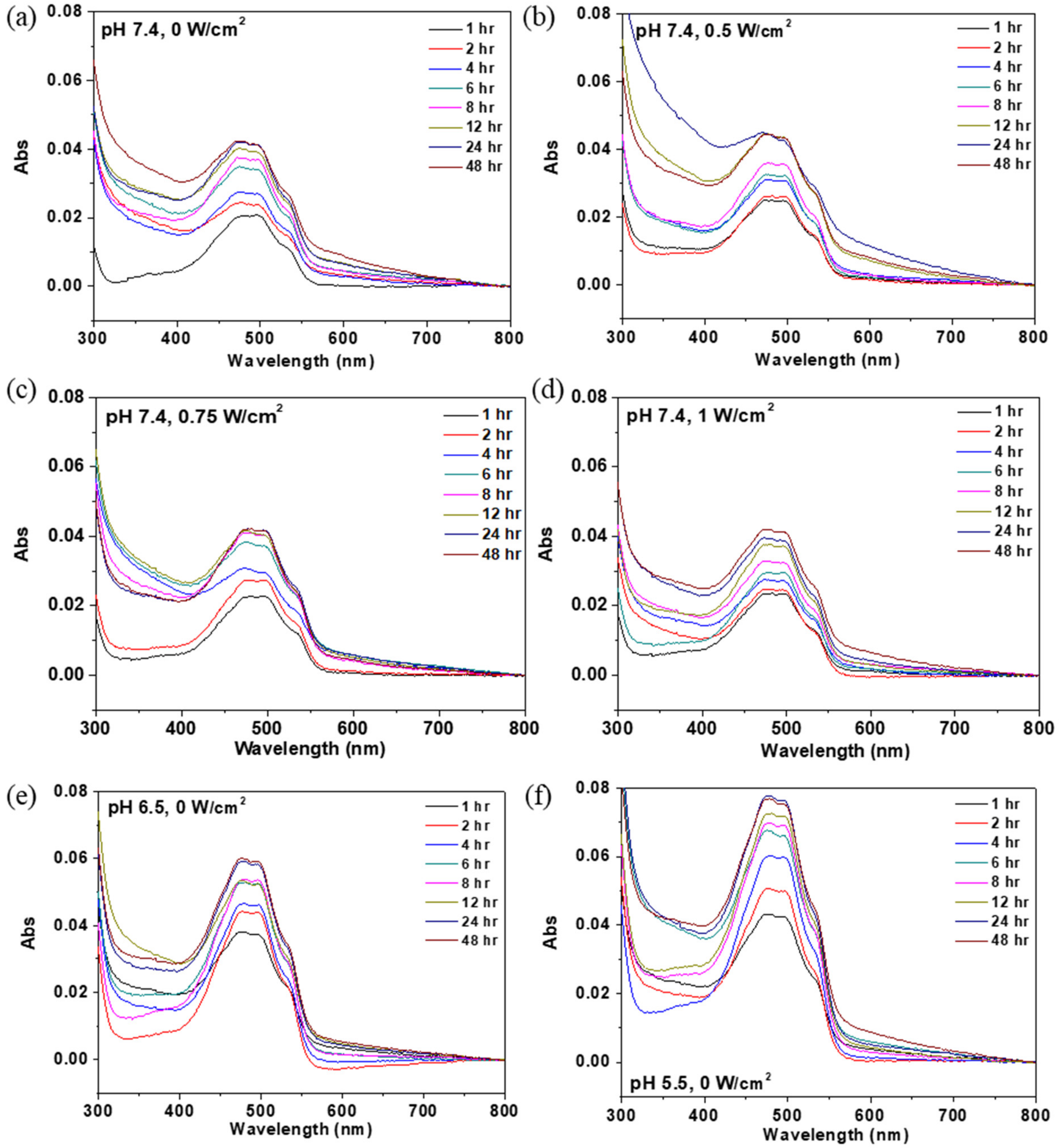

Figure S19. UV-Vis spectra of DOX-loaded D2 polymeric micelle for in vitro drug release under varying pH values and ultrasound power densities at different time points.

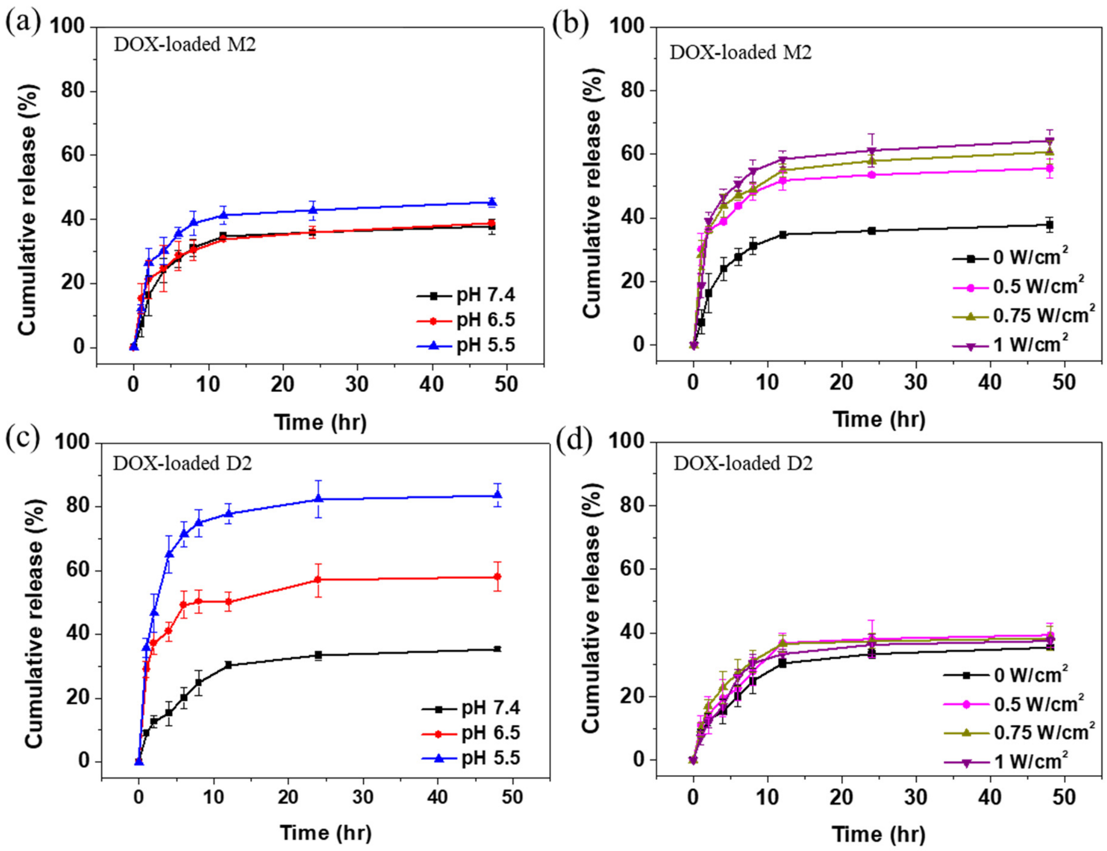

Figure S20. *In vitro* drug release of DOX-loaded polymeric micelles at different time points. (a) DOX-loaded M2 polymeric micelle at different pH values without ultrasound irradiation. (b) DOX-loaded M2 polymeric micelle at pH 7.4 with different ultrasound power densities. (c) DOX-loaded D2 polymeric micelle at different pH values without ultrasound irradiation. (d) DOX-loaded D2 polymeric micelle at pH 7.4 with different ultrasound power densities.

Table S1. GPC analysis of $mPEG_X$-CTA

$mPEG_X$-CTA	M_n[a]	M_n[b]	M_w[b]	ĐM[b]	M_n[c]	M_w[c]	ĐM[c]
$mPEG_{45}$	2000	2449	2565	1.05	2904	3044	1.05
$mPEG_{45}$-CTA	2397	2886	2974	1.03	3598	3814	1.06
$mPEG_{90}$	4000	4146	4342	1.05	6079	6349	1.05
$mPEG_{90}$-CTA	4377	4522	4926	1.09	6695	7147	1.07

[a] M_n of polymers were estimated by NMR.

[b] M_n, M_w, and ĐM of polymers were determined by GPC in water

[c] M_n, M_w, and ĐM of polymers were determined by GPC in THF

Table S2. DLE and DLC of DOX-loaded polymeric micelles

Polymeric micelle	Concentration of loaded DOX （μg/mL）	DLE （wt%）	DLC （wt%）
**DM2**	45.8	32.1	6.4
**DM4**	39.3	29.5	5.9
**M2**	36.6	27.4	5.5
**D2**	44.9	31.4	6.3

Table S3. Drug release data fitting with a logarithmic model

Sample	Condition	Slope	Intercept	R^2
**DOX-loaded DM2**	0 W/cm², pH 7.4	6.07	9.6741	0.9635
	0.5 W/cm², pH 7.4	6.5702	20.276	0.9384
	0.75 W/cm², pH 7.4	8.7169	22.119	0.9894
	1 W/cm², pH 7.4	10.025	24.826	0.9569
	0 W/cm², pH 6.5	12.064	24.261	0.9043
	0 W/cm², pH 5.5	12.986	35.687	0.8359
	1 W/cm², pH 7.4	10.025	24.826	0.9569
	1 W/cm², pH 6.5	8.8045	37.054	0.974
	1 W/cm², pH 5.5	12.639	32.967	0.9761
**DOX-loaded DM4**	0 W/cm², pH 7.4	8.0282	8.4038	0.9808
	0.5 W/cm², pH 7.4	8.1513	28.23	0.929
	0.75 W/cm², pH 7.4	9.399	31.268	0.9449
	1 W/cm², pH 7.4	11.478	34.371	0.8934
	0 W/cm², pH 6.5	13.458	28.793	0.9272
	0 W/cm², pH 5.5	12.98	36.04	0.9028
	1 W/cm², pH 7.4	11.478	34.371	0.8934
	1 W/cm², pH 6.5	10.03	41.199	0.949
	1 W/cm², pH 5.5	10.061	53.698	0.7736

The drug release data were fitted to a logarithmic model of the form $Q(t) = a \times ln(time) + b,$ where:

- Q(t) represents the cumulative drug release percentage at the time,
- a is the slope indicating the rate of drug release,
- b is the intercept representing the initial drug release percentage.
